# Rapid Quantification of Pharmaceuticals via ^1^H Solid-State NMR Spectroscopy

**DOI:** 10.1021/acs.analchem.2c02905

**Published:** 2022-11-23

**Authors:** Y. T. Angel Wong, Ruud L. E. G. Aspers, Marketta Uusi-Penttilä, Arno P. M. Kentgens

**Affiliations:** † Institute for Molecules and Materials, Radboud University, Heyendaalseweg 135, 6525 AJNijmegen, The Netherlands; ‡ Aspen Oss B.V., Kloosterstraat 6, 5349 ABOss, The Netherlands

## Abstract

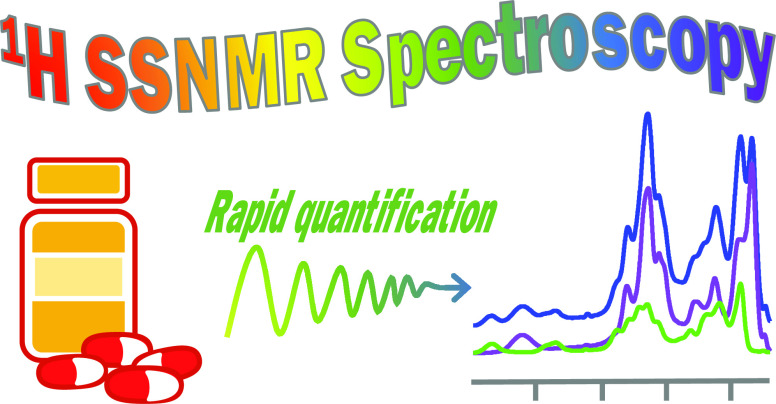

The physicochemical properties of active pharmaceutical ingredients (APIs) can depend on their solid-state forms. Therefore, characterization of API forms is crucial for upholding the performance of pharmaceutical products. Solid-state nuclear magnetic resonance (SSNMR) spectroscopy is a powerful technique for API quantification due to its selectivity. However, quantitative SSNMR experiments can be time consuming, sometimes requiring days to perform. Sensitivity can be considerably improved using ^1^H SSNMR spectroscopy. Nonetheless, quantification via ^1^H can be a challenging task due to low spectral resolution. Here, we offer a novel ^1^H SSNMR method for rapid API quantification, termed CRAMPS–MAR. The technique is based on combined rotation and multiple-pulse spectroscopy (CRAMPS) and mixture analysis using references (MAR). CRAMPS–MAR can provide high ^1^H spectral resolution with standard equipment, and data analysis can be accomplished with ease, even for structurally complex APIs. Using several API species as model systems, we show that CRAMPS–MAR can provide a lower quantitation limit than standard approaches such as fast MAS with peak integration. Furthermore, CRAMPS–MAR was found to be robust for cases that are inapproachable by conventional ultra-fast (i.e., 100 kHz) MAS methods even when state-of-the-art SSNMR equipment was employed. Our results demonstrate CRAMPS–MAR as an alternative quantification technique that can generate new opportunities for analytical research.

## Introduction

Active pharmaceutical ingredients (APIs) can exist as multiple solid-state forms such as polymorphs,^1,2^ salts,^2,3^ and hydrates.^4^ Since different forms can exhibit different physicochemical properties, drug development and manufacturing requires careful control over the desired form.^1−4^ For instance, APIs are often produced as salts to increase solubility and dissolution rates.^2,3^ Undesired conversion to the neutral form can compromise the drug performance. APIs can also display extensive polymorphism, and the identity of the polymorph can alter the solubility, dissolution rate, and bioavailability of the drug.^1,2^ Given the complex interplay between the solid-state forms and API performance, identification and quantification of API forms is crucial for pharmaceutical research.

API characterization can be a challenging task. The structural similarities between solid-state forms and the heterogeneous composition of a pharmaceutical product can obscure analysis.^5−9^ A promising method for API quantification is solid-state nuclear magnetic resonance (SSNMR) spectroscopy.^10−12^ SSNMR spectroscopy is a non-destructive technique that can be applied on crystalline and amorphous materials.^13−20^ Moreover, SSNMR spectroscopy can provide high selectivity. Structurally similar API forms can be distinguished via SSNMR spectroscopy.^5−8,21−28^ In some cases, SSNMR spectroscopy can outperform powder X-ray diffraction.^5−7^ For example, the isomorphous solvate hydrates of finasteride were found to give comparable powder X-ray diffractograms but clearly distinct ^13^C SSNMR spectra.^5^ Similar observations were made for the polymorphs of neotame.^6^ Furthermore, by judiciously choosing which nuclei to examine, API signals can be selectively detected. This has been demonstrated using nuclei such as ^35^Cl and ^19^F, where spectra of APIs were obtained without interferences from excipients.^8,16,29−31^ In contrast to many analytical techniques, SSNMR spectroscopy is inherently quantitative since the peak area is directly proportional to the number of spins. Thus, for some SSNMR experiments, quantification can be performed without calibration.^18,19,21,22,31−33^

^13^C SSNMR spectroscopy is commonly employed for API quantification since carbons are ubiquitous in APIs.^15,20,22,25,33,34^ Nonetheless, ^13^C SSNMR experiments can be time consuming due to the low natural abundance of ^13^C, and spectra with sufficient signal-to-noise ratio (SNR) can require days to aquire.^21,35^ While ^13^C isotopic enrichment can decrease experimental time,^26^ isotopic labeling can be expensive and labor intensive. Furthermore, isotopic labeling is incompatible with quality checks during manufacturing as pharmaceutical end products are not isotopically enriched. An alternative approach to reduce experimental time is ^1^H SSNMR spectroscopy since ^1^H has a significantly higher natural abundance and gyromagnetic ratio than ^13^C.^21,35^ For example, Hirsh et al. showed that ^1^H quantification can decrease the experimental time by one to three orders of magnitude compared to conventional ^13^C SSNMR approaches.^35^ However, acquiring ^1^H SSNMR data adequate for quantification can be a challenging task as well. Strong ^1^H–^1^H dipolar coupling interactions are often present in solids, resulting in broad linewidths and a severe decline in spectral resolution. A common method to boost spectral resolution is fast or ultra-fast magic-angle spinning (MAS). In this method, the sample is spun at speeds greater than 40 kHz, with 120 kHz being the maximum frequency currently commercially available.^36,37^ Fast or ultra-fast MAS has been applied on various APIs to increase ^1^H spectral resolution,^14,21,30,35,38−42^ with some using spinning speeds above 100 kHz.^38,39,41,42^ The boost in spectral resolution can facilitate quantitative studies. For instance, Li et al. employed an external magnetic field strength (*B*_o_) of 9.40 T (ν_o_(^1^H) = 400 MHz) and showed that ^1^H spectral resolution of pioglitazone hydrochloride (PioHCl) salts, an anti-diabetes drug, can be drastically improved at high spinning speeds .^21^ At 60 kHz, the ^1^H SSNMR spectra contain adequate resolution for quantification. Nonetheless, fast and/or ultra-fast MAS requires specialized probes that are costly. Another method to enhance ^1^H resolution is via combined rotation and multiple-pulse spectroscopy (CRAMPS),^43−46^ such as decoupling using mind-boggling optimization (DUMBO).^47,48^ These experiments rely on both sample spinning and radiofrequency (RF) pulses to reduce ^1^H linewidths. The combination allows for a slower spinning speed (e.g., ca. 12 kHz);^43^ thus, these experiments can be performed with readily available standard equipment. Furthermore, larger-diameter rotors can be employed at slower spinning speeds. This enables a higher volume of sample to be analyzed, which can provide a potentially better SNR. For instance, the larger rotors available for a spinning speed of 12 kHz (e.g., 3.2 mm rotor diameter) can boost the SNR by an approximate factor of 3 compared to the smaller rotors available for 60 kHz MAS (e.g., 1.2, or 1.3 mm rotor diameter). Additionally, CRAMPS can provide better resolution. Several works have shown that for spinning speeds up to 80 kHz, CRAMPS can yield higher resolution than MAS alone.^49−54^ Furthermore, Leskes et al. demonstrated that CRAMPS at slower spinning speeds can still give narrower signals than fast MAS.^54^ For glycine, CRAMPS spectra recorded at 35 to 55 kHz all provided higher resolution than fast MAS experiments at 65 kHz.

Besides challenges with data acquisition, ^1^H SSNMR data analysis can also be difficult. Several approaches can be used to quantitatively analyze SSNMR data, such as relaxation,^15,18^ chemometric,^17,22,55^ peak-integration,^21,35^ and reference-based methods.^19,33^ To the best of our knowledge, previous quantitative ^1^H SSNMR studies of APIs have only been performed with peak-integration-based methods.^21,35^ However, the main disadvantage of this approach is it requires well-resolved signals. Even when advanced ^1^H SSNMR techniques such as ultra-fast MAS and CRAMPS are used, the resulting spectra can still have inadequate resolution. The spectral congestion can render data analysis a challenging, if not impossible, task. Recently, Stueber and Dance showed that quantitative ^13^C and ^19^F SSNMR data can be processed via mixture analysis using references (MAR).^33^ In MAR, the mixture spectrum is fitted as a linear combination of the pure component spectra. The corresponding results provide insights into the mixture composition. MAR can offer the same level of accuracy as conventional peak-integration-based methods but does not require baseline resolution for an effortless analysis. Thus, MAR can be beneficial for quantitative ^1^H SSNMR studies, where the corresponding spectra are often highly congested.

Despite the benefits of MAR, analyzing CRAMPS spectra via MAR can be non-trivial. Since MAR employs pure component spectra to fit the mixture spectrum,^33^ the pure component spectra must resemble the constituents of the mixture spectrum for an accurate fit. For CRAMPS, spectral features such as resolution, lineshapes, and absolute peak positions are dictated by chemical shift scaling factors.^56,57^ To successfully apply MAR on CRAMPS spectra, any discrepancies between the scaling factors of the mixture and pure component spectra must be accounted for during data processing. However, standard CRAMPS data processing methods, such as obtaining the scaling factor via an external reference,^43^ assume identical scaling factors between datasets. These methods can therefore overlook differences between the scaling factors of the mixture and pure component spectra. As such, the standard methods are inadequate for MAR, and a different data processing approach is required.

Here, we present an SSNMR approach for quantifying solid-state forms of APIs, termed CRAMPS–MAR. In this method, data acquisition is performed with ^1^H CRAMPS, and analysis is accomplished with MAR. CRAMPS alleviates the need for specialized equipment without sacrificing spectral resolution, while MAR enables an effortless analysis of complex spectra. Thus, the combination, CRAMPS–MAR, allows for a rapid, accurate, and straightforward API quantification procedure. Our results demonstrate that CRAMPS can provide higher ^1^H spectral resolution than ultra-fast MAS, even when the ultra-fast MAS experiments are performed at a significantly higher *B*_o_. Moreover, we have developed CRAMPS data processing procedures to compensate for variations between the scaling factors of the pure component and mixture spectra, allowing for an accurate MAR fit. Using mixtures containing PioHCl and pioglitazone (Pio), we demonstrate that CRAMPS–MAR outperforms previously reported results from fast MAS with peak integration.^21^ To show that our method is applicable for structurally complex systems, we studied a polymorphic blend of a pharmaceutically relevant steroid,^58^ Org OD 14, also known as 7αMNa or tibolone. Although the structural complexity of steroids typically leads to severely congested SSNMR spectra, CRAMPS–MAR was able to provide accurate quantification results. Analogous quantification was found to be impractical with peak-integration-based methods even when the data was obtained using state-of-the-art ultra-fast MAS equipment.

## Experimental Section

### Sample Preparation

Pio (99% purity) was purchased from Doug Discovery. PioHCl (> 98% purity) was obtained from Tokyo Chemical Industry (TCI). Both Pio and PioHCl were used as purchased. Form I and form II polymorphs of Org OD 14 (Org-I and Org-II, respectively) were provided by Aspen Oss B.V. The chemical structures of Pio, PioHCl, and Org OD 14 are shown in Figure S1.

For the Pio/PioHCl systems, seven samples ranging from 5 to 97 weight percent (wt %) Pio were prepared. For the Org-I/-II sample, a bicomponent mixture containing 69.9 weight percent Org-I was made. Additional sample preparation details are given in the Supporting Information.

### SSNMR Experiments

For samples containing Pio and/or PioHCl, all experiments were performed using a Varian VNMRS spectrometer at a *B*_o_ of 9.40 T (ν_o_(^1^H) = 400 MHz), a Varian 3.2 mm T3 HXY probe, and large-volume rotors. The samples were restricted to the center third of the rotors. The exact sample weights inside the rotors were determined using an analytical balance and are ca. 30 mg. The ^1^H CRAMPS experiments were performed at 11.1 kHz MAS using a super-cycled^59^ windowed DUMBO (wDUMBO) pulse sequence with DUMBO-1 coefficients^47^ and a five-point phase ramp.^60^ The experimental temperature was regulated to 21 °C to avoid variations in Boltzmann distributions between the pure component and mixture spectra. The sample spinning speed, probe tuning frequency, and transmitter offset frequency (TOF) were optimized for resolution (see the Supporting Information for details). Moreover, CRAMPS spectra can suffer from artifacts such as rotary resonance frequency (RRF) lines. The positions of RRF lines are determined by MAS and CRAMPS cycle frequencies and are independent of the TOF.^61,62^ Since RRF lines do not contain any weight percent information, overlaps between sample signals and RRF lines were minimized by carefully adjusting the TOF without decreasing spectral resolution.^43^ The absolute chemical shift scaling factors were determined using the NH_3_ and CH_2_ signals of α-glycine at 8.0 and 2.6 ppm, respectively.^63^ The ^1^H chemical shifts were referenced using α-glycine (δ_iso_(CH_2_) = 2.6 ppm).^63^

For the Org OD 14 samples, the ^1^H CRAMPS experiments were performed using the same setup and/or conditions as the Pio and PioHCl samples. Ultra-fast MAS ^1^H SSNMR experiments were performed on a Varian VNMRS spectrometer at a *B*_o_ of 19.96 T (ν_o_(^1^H) = 850 MHz) with a Bruker 0.7 mm ultra-fast MAS probe. The spectra were acquired with a one-pulse experiment at 100 kHz MAS. The ^1^H chemical shifts were referenced using α-glycine (δ_iso_(NH_3_) = 8.0 ppm)^63^ as a secondary reference.

The ultra-fast MAS spectra were fully processed using ssNake.^64^ For the CRAMPS spectra, ssNake^64^ was employed for truncation, zero-filling, Fourier transform, and phasing. The pure component and mixture spectra were first phased independently to absorption mode. The spectra were then overlaid for a visual inspection. If the spectra were out of phase with each other, the zeroth- and/or first-order phasing was adjusted until the spectra are in-phase. MAR processing was performed using an in-house MATLAB^65^ script. Additional experimental and data processing details and the CRAMPS–MAR processing MATLAB scripts are provided in the Supporting Information.

## Results and Discussion

### Description of CRAMPS–MAR

In the MAR method, the spectrum for a mixture with N components is represented as a weighted linear sum of the pure component spectra (*p*_1_, *p*_2_, ..., *p_N_*).^33^ The mixture spectrum, *m*, can therefore be expressed as
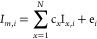
where *I*_*m*,*i*_ denotes the spectral intensity of *m* at frequency *i*, *I*_*x*,*i*_ denotes the spectral intensity of *p_x_* at frequency *i*, c_*x*_ is the weighting coefficient for *p_x_*, and *e_i_* represents the residual error at frequency *i* (e.g.*,* noise). By fitting *I_m_* with *I_x_* (*x* = 1 to N) via least-squares, *c_x_*, and therefore the mixture composition, can be determined.

Figure S2 illustrates the complete workflow of CRAMPS–MAR. To obtain accurate quantification results, the appearance of the pure component spectra must represent the components in the mixture spectrum. Therefore, the spectra must be in-phase with each other. Significant deviation in phasing will give inaccurate CRAMPS–MAR results (see the Supporting Information for a detailed discussion). Moreover, for ^1^H CRAMPS experiments, spectral features such as resolution, lineshape, and peak positions are dictated by chemical shift scaling factors, which are sensitive to experimental settings.^56,57^ These settings include the sample position relative to the RF coil, probe tuning frequency, decoupling RF field strength, and TOF.^56,57^ We found that the spectral resolution and lineshapes of the pure and mixture spectra are comparable if the sample positions are restricted to the center third of the rotors, and the spectra are recorded using the same probe tuning frequency, RF field strength, and TOF. However, an offset in peak positions was still observed due to small differences in the scaling factors (Figure S7).^43,56,57^ Thus, solely regulating the experimental settings is insufficient to standardize the scaling factors between different spectra. We have developed additional data processing procedures to account for the variations in scaling factors. These procedures involve scaling and aligning the pure component spectra to the mixture spectrum. The scaling factor must also be applied on the *y*-axis for the peak areas to remain the same, allowing for quantitative analysis. An in-depth description on the scaling procedure is given in the Supporting Information.

Due to the scaling, the data points of each spectrum will occur at different frequencies. Consequently, the spectra must be binned. Excessive binning will degrade the apparent spectral resolution; thus, minimal binning should be conducted. A visual comparison between the pre- and post-binned spectra can be employed as a gauge. If the apparent spectral resolution of the post-binned spectrum is comparable to the pre-binned spectrum, then the binning is adequate. Practically, spectral resolution will be preserved upon binning if an additional zero-fill is performed during FID processing, and the Fourier transformed data is binned to at most two data points per bin. A detailed discussion on binning, and how it influences fit results and analysis time, is provided in the Supporting Information.

To obtain the mixture composition with MAR, the spectra must be normalized to the sample weight and number of scans (assuming constant temperature and *B*_o_). A least-square fitting of *I_m_* using *I_x_* (*x* = 1 to *N*) will then provide the mixture composition in weight percent. A weight of 0 should be assigned to the RRF lines in the least-square fit region as they do not contain any weight percent information.

### Quantification of Pio/PioHCl Mixtures

**Figure 1:**
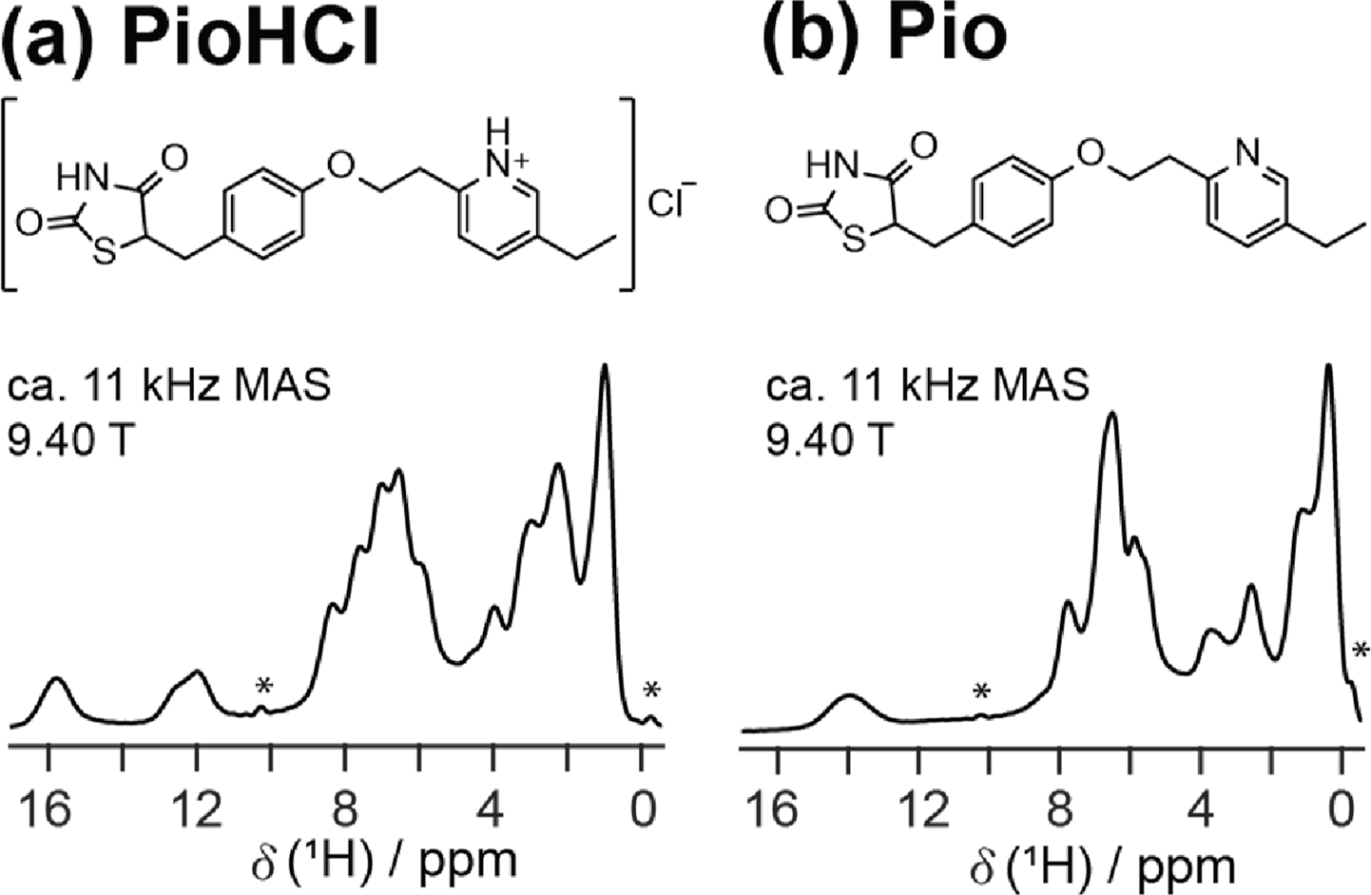
The ^1^H wDUMBO SSNMR spectra of pure (a) PioHCl and (b) Pio, and the corresponding chemical structures. Asterisks (*) denote RRF lines.

The ^1^H wDUMBO spectra of pure Pio and PioHCl are given in Figure 1. For both samples, the highest spectral resolution is observed in the region of ca. 11 to 17 ppm. This region contains ^1^H signals corresponding to nitrogen-bound protons, i.e., N–H and N·HCl.^21^ In the aromatic and aliphatic regions (ca. 0 to 9 ppm), the spectra are more crowded. Nonetheless, distinct spectral features can still be observed, and the resolution is notably higher than spectra recorded at the same *B*_o_ (9.40 T) using fast MAS at 60 kHz (figure 3 of ref (21)). The increase in resolution is the most apparent in the 6 to 10 ppm region of Pio and the 0 to 10 ppm region of PioHCl. A similar behavior has been observed for glycine, where wDUMBO experiments at moderate spinning speeds (35 to 55 kHz) provided higher spectral resolution than MAS experiments at 65 kHz.^54^ The resolution of MAS spectra can be further increased via ultra-fast MAS (i.e., spinning speed ≥100 kHz).^66,67^ Nonetheless, the ^1^H linewidths do not scale linearly with spinning speeds (see figure 5 of ref (66)). For example, the ^1^H linewidths of l-histadine·HCl·H_2_O were only reduced by a maximum of ca. 37% when the spinning speed was increased from 60 to 120 kHz, which is the current limit of commercial equipment.^67^ As such, we expect the wDUMBO results to still compare favorably to those obtained using ultra-fast MAS. This was indeed observed for Org OD 14, where the wDUMBO spectra displayed a higher resolution than those acquired at a spinning speed of 100 kHz (vide infra).

**Figure 2:**
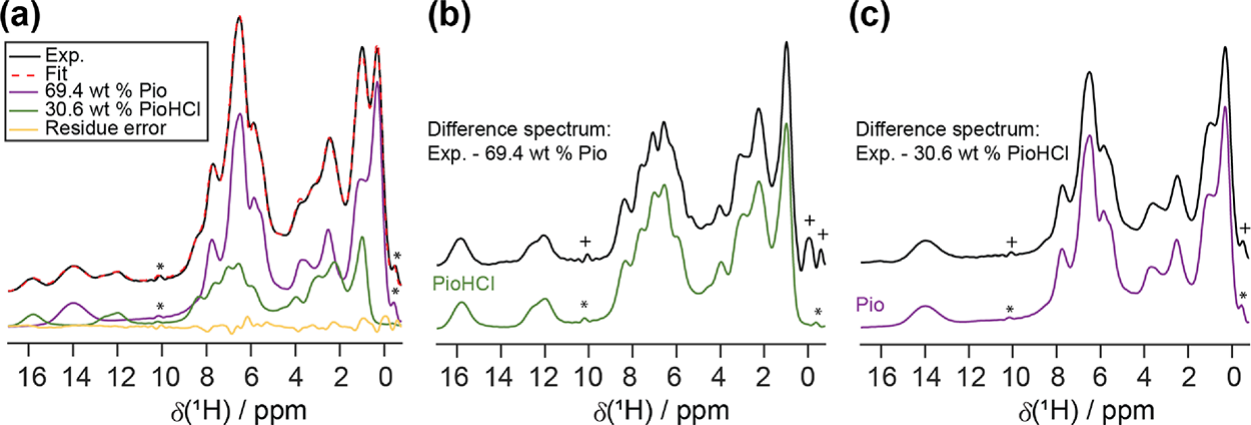
(a) The experimental ^1^H wDUMBO spectrum (black trace) and the corresponding CRAMPS–MAR fit (red dotted trace) for Pio 70%. The individual fit components (Pio: purple trace, PioHCl: green trace, residue error: yellow trace) are also provided. (b) The difference spectrum (black trace) obtained by subtracting the CRAMPS–MAR-predicted Pio contribution from the experimental spectrum, and the pure PioHCl spectrum (green trace). (c) The difference spectrum (black trace) acquired by removing the CRAMPS–MAR-predicted PioHCl contribution from the experimental spectrum, and the pure Pio spectrum (purple trace). Asterisks (*) denote RRF lines, and crosses (+) denote artifacts from applying 0 weight on the RRF lines during fitting.

We have quantified seven bicomponent samples containing 5 to 97 wt % Pio using CRAMPS–MAR. The exact sample compositions and the CRAMPS–MAR results are given in Table S8. Figure 2 shows the results for Pio 70%. The CRAMPS–MAR fit agrees well with the experimental spectrum, and the residue spectrum does not resemble sample signals. Moreover, if the pure component spectra were properly scaled and aligned to the mixture spectrum, the difference spectrum obtained from *m* – *c*_1_*p*_1_ should mimic *p*_2_. Figure 2 shows that the spectrum of *m* – *c*_PIO_*p*_PIO_ resembles the pure PioHCl spectrum while the spectrum of *m* – *c*_PIOHCl_*p*_PIOHCl_ looks like the pure Pio spectrum. Thus, the pure component spectra were adequately scaled and aligned. Comparable fit quality was found for all other samples, and the respective spectra are provided in Figure S12.

**Figure 3:**
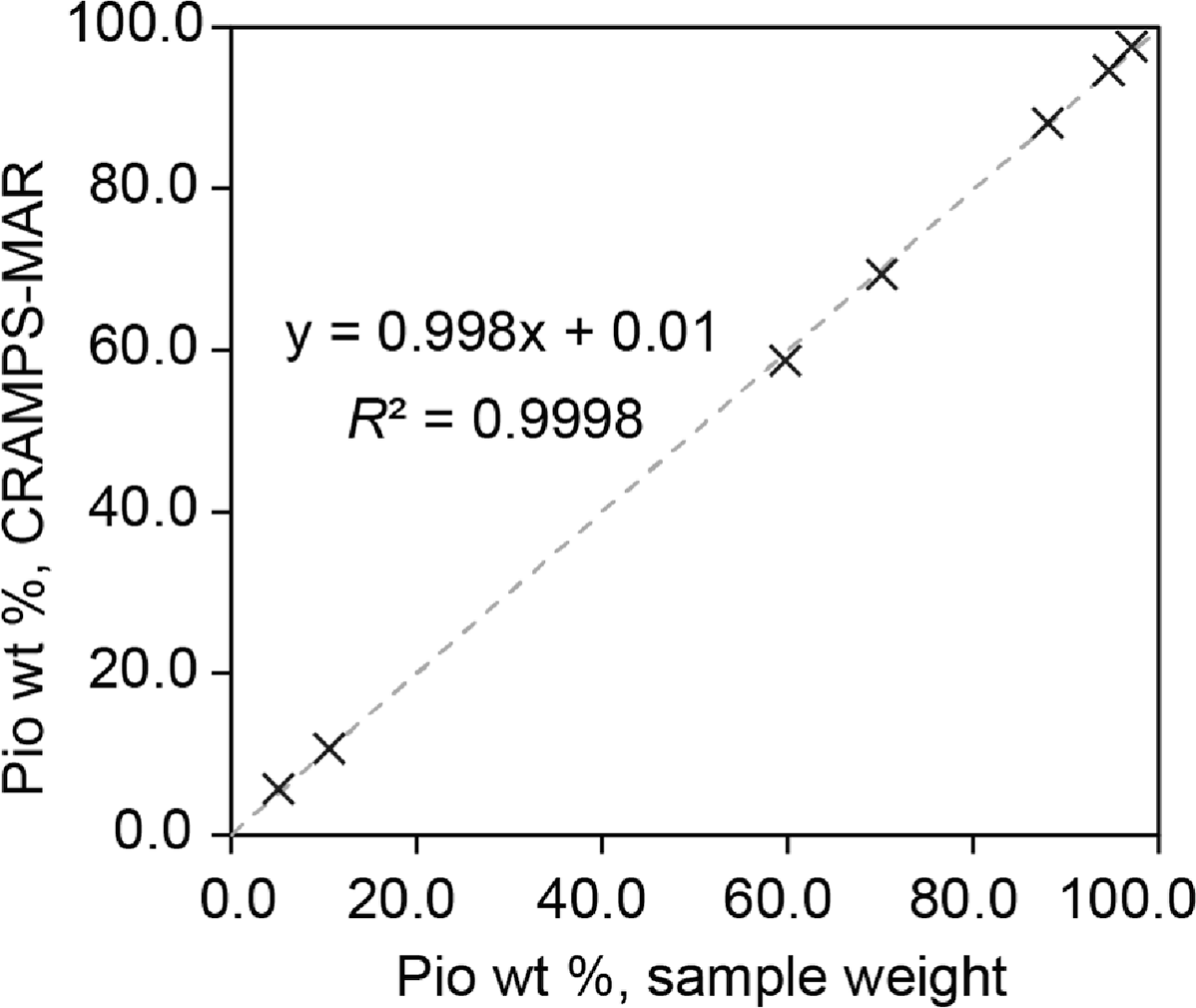
Correlation between the Pio weight percent (Pio wt %) determined by sample weight and CRAMPS–MAR. The line of best fit is also provided (gray dotted line). Errors bars are not shown but are provided in Table S8.

To further evaluate the CRAMPS–MAR results, the weight percent values from CRAMPS–MAR are plotted in Figure 3 against the sample weight values. A good agreement is found, with a coefficient of determination (*R*^2^) value greater than 0.999. Moreover, the CRAMPS–MAR values are within ±1 wt % of the sample weight values (Table S8). This is reflected by the correlation plot (Figure 3), which displays a slope of near unity (0.998) and an intercept of ca. 0. The accuracy of our CRAMPS–MAR results compare favorably with those reported for MAR-based ^13^C and ^19^F SSNMR studies (slope = 0.99, intercept = 0.24 to 0.30).^33^

Even though previous studies have shown that ^13^C and/or ^19^F SSNMR spectroscopy can provide accurate MAR results, the experiments are time consuming since each spectrum can require hours to record.^33^ On the other hand, ^1^H SSNMR experiments can be significantly faster due to the high gyromagnetic ratio and natural abundance of ^1^H. This is demonstrated using Pio 10% (Figure S13). We found that even at this low weight percent value, mixture spectra suitable for accurate quantification can be acquired in 6 min. Moreover, CRAMPS–MAR analysis can be accomplished within seconds (see the Supporting Information). It should be noted that the experimental time was not limited by the SNR but by the minimal number of scans required for complete phase cycling. Furthermore, we used a recycle delay that is five times longer than the longitudinal relaxation time (*T*_1_) values. The experimental time could be further decreased if the truMAR method, which uses a recycle delay of 1.2 times the *T*_1_ values, is employed.^33^ As shown by Stueber and Dance using ^13^C SSNMR spectroscopy, a ca. 75% reduction in experimental time can be obtained via truMAR.^33^

**Figure 4:**
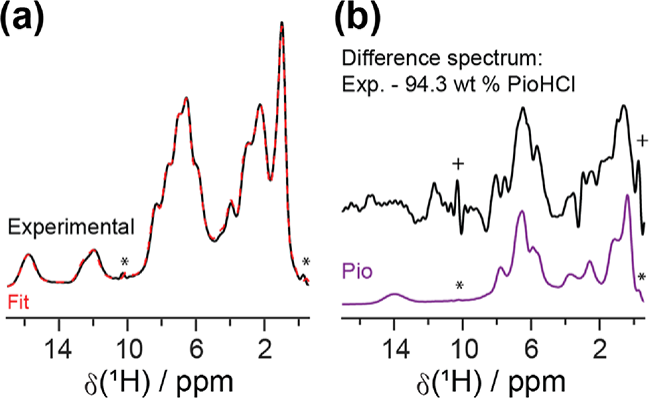
(a) The ^1^H wDUMBO spectrum (black trace) of Pio 5% with the corresponding CRAMPS–MAR fit (red dotted trace). (b) The difference spectrum (black trace) generated by subtracting the CRAMPS–MAR-predicted PioHCl contribution from the experimental spectrum. The pure Pio spectrum (purple trace) is also provided for reference. Asterisks (*) denote RRF lines, and crosses (+) denote artifacts from applying 0 weight on the RRF lines during fitting.

Pio/PioHCl bicomponent mixtures have been previously quantified using fast MAS experiments with a spinning speed of 60 kHz. Two-dimensional correlation experiments were used to assign the 11 to 17 ppm signals. The corresponding peaks in the one-dimensional spectra were integrated and normalized based on the signal identities. Using this method, samples containing ca. 10 to 90 wt % Pio were quantified.^21^ Here, we accurately quantified mixtures with ca. 5 and 97 wt % Pio without signal assignments, allowing for a less labor- and time-intensive analysis procedure. Furthermore, our CRAMPS–MAR results (5 to 97 wt % Pio) exceed the limits demonstrated by fast MAS with signal integration (10 to 90 wt % Pio). The success of our methods can be attributed to two main factors. First, CRAMPS experiments can offer superior spectral resolution compared to fast MAS experiments. For MAR, high resolution provides more spectral features for the least-square fit, resulting in a more sensitive quantification. Second, MAR has higher tolerance for peak overlap than the signal integration method. In the signal integration approach, accurate quantification requires well-resolved peaks. Thus, for Pio/PioHCl, only the ca. 11 to 17 ppm region is usable (Figure 1). However, at lower weight percent, the peaks in this region can become undetectable, making quantification by signal integration unfeasible. This is seen in the Pio 5% spectrum (Figure 4), where the Pio signal at ca. 14 ppm is no longer observed. On the other hand, the MAR method is based on “fingerprinting” and can therefore be applied on highly congested regions. Hence, the 0 to 10 ppm region can be analyzed. Figure 4 shows that the CRAMPS–MAR method provides an excellent fit. The difference spectrum of *m* – *c*_pioHCl_*p*_pioHCl_ looks similar to pure Pio in the 0 to 10 ppm range, verifying that this spectral region contains useful quantitative data. Thus, by utilizing valuable spectral regions that are inaccessible to the signal integration approach, the CRAMPS–MAR method outperforms the signal integration method.

### Quantification of Org OD 14

To the best of our knowledge, ^1^H SSNMR-based quantification of steroids has yet to be attempted, likely due to the structural complexity of these compounds. To further investigate the limits of CRAMPS–MAR, we have quantified a steroid, Org OD 14, using a sample (Org-I 70%) that contains 69.9 wt % of polymorph Org-I and 30.1 wt % of polymorph Org-II. Previous studies have shown that Org-I/-II mixtures can be quantified via natural abundance ^13^C SSNMR spectroscopy.^58^ However, this can be time consuming. For example, in our experience, it can take up to a week to acquire quantifiable ^13^C SSNMR data for Org-I/-II samples with 1 wt % Org-II, even when sensitivity enhancement techniques such as ^1^H →^13^C cross-polarization are employed.

**Figure 5:**
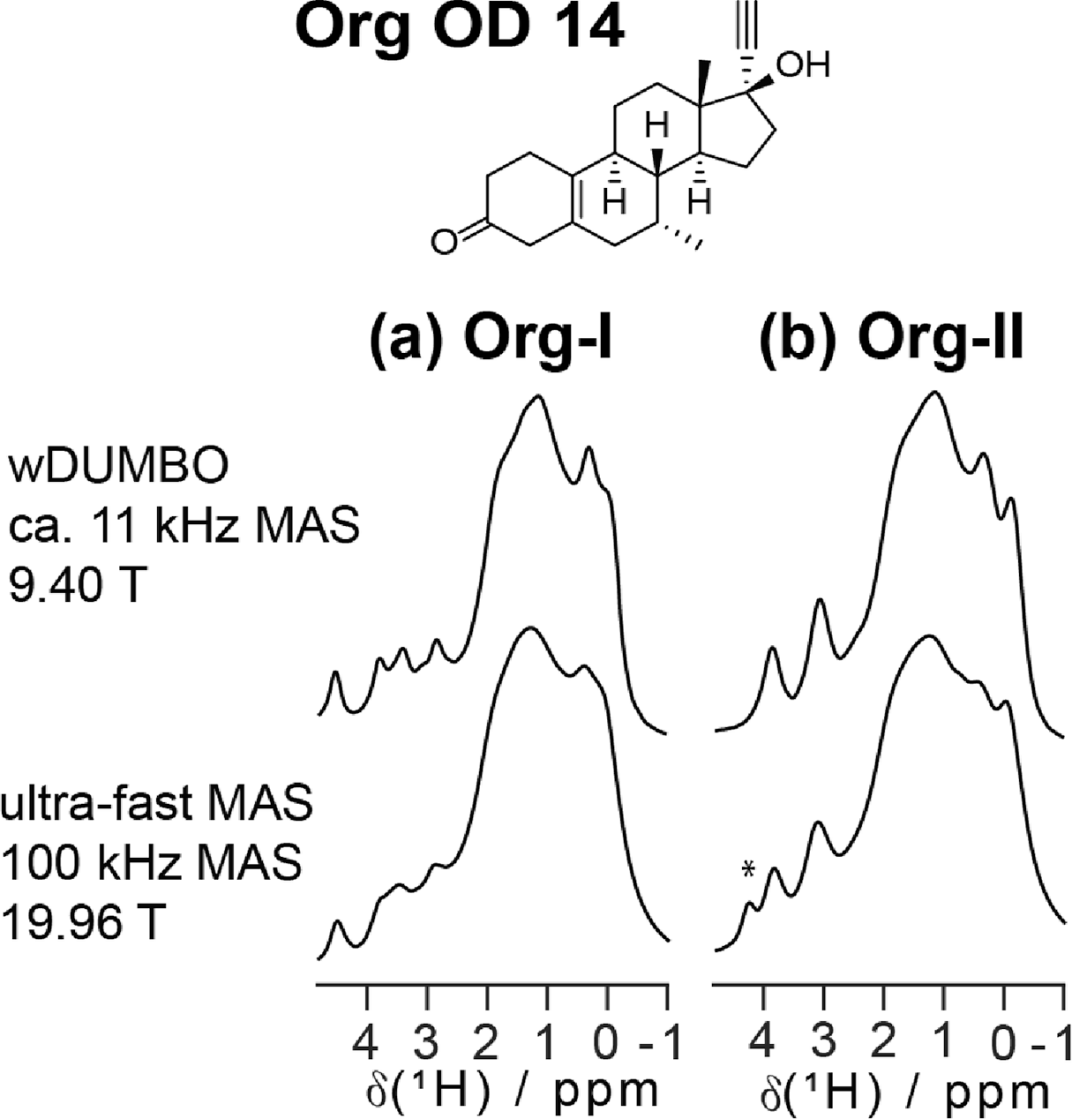
The chemical structure of Org OD 14, and the ^1^H SSNMR spectra of (a) Org-I and (b) Org-II recorded using wDUMBO (top) and ultra-fast MAS (bottom) experiments. Asterisk (*) denotes impurity.

Figure 5 shows the ^1^H wDUMBO spectra of Org-I and Org-II. Based on the number of crystallographically inequivalent hydrogens in the Org-I and Org-II crystal structures, 56 and 28 ^1^H SSNMR signals, respectively, can be expected.^68,69^ However, only ca. six and five distinct spectral features were observed in our Org-I and Org-II spectra, respectively. The spectral congestion is attributed to the complex chemical structure of Org OD 14 and the presence of strong ^1^H–^1^H dipolar interactions. For comparison, we have also acquired ^1^H SSNMR spectra of Org-I and Org-II using an ultra-fast MAS speed of 100 kHz and an ultra-high *B*_o_ of 19.96 T (Figure 5). Remarkably, the ^1^H wDUMBO spectra display higher resolution even though they were acquired with a substantially slower spinning speed (ca. 11 kHz) and at ca. half the *B*_o_ value (9.40 T). This is the most notable in the ca. 2 to 4 ppm region of Org-I, where the wDUMBO spectra present more detailed spectral features. The increase in resolution is also reflected by the full width at half maximum (FWHM) value of the well-resolved Org-I signal at 4.5 ppm. An FWHM of 350 Hz (0.41 ppm) was observed at 100 kHz spinning, while an FWHM of 140 Hz (0.35 ppm, rescaled linewidth) was found using wDUMBO. Further increasing the spinning speed to the currently commercially available limit of 120 kHz will have negligible influence as ^1^H linewidths do not scale linearly with spinning speed.^66^ To boost the spectral resolution in a meaningful manner, the spinning speed needs to be increased substantially. Nonetheless, even when such technology is available, the rotors will be impractically small, potentially resulting in sample preparation and SNR issues.

**Figure 6:**
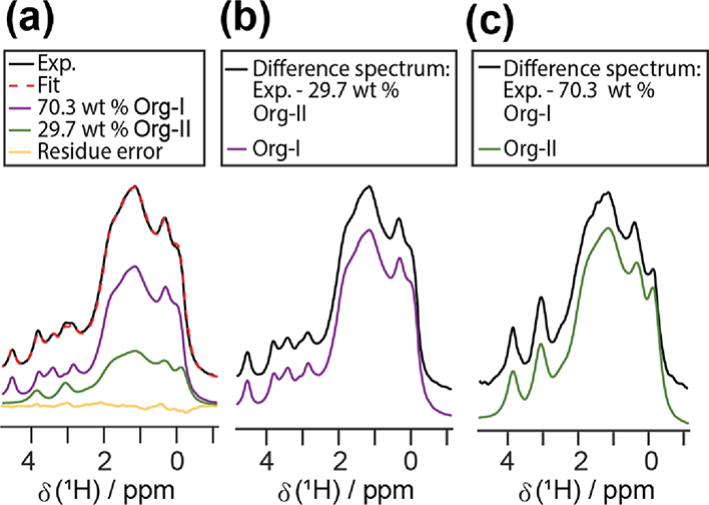
(a) The ^1^H wDUMBO spectrum (black trace) and the CRAMPS–MAR fit (dotted red trace) for Org-I 70%. The individual fit components are also provided (Org-I: purple trace, Org-II: green trace, residue error: yellow trace). (b) The difference spectrum (black trace) calculated by subtracting the CRAMPS–MAR-predicted Org-II contribution from the experimental spectrum. The pure Org-I spectrum is also provided for reference (purple trace). (c) The difference spectrum (black trace) obtained by deducting the CRAMPS–MAR-predicted Org-I contribution from the experimental spectrum. The pure Org-II is also given (green trace).

The pure component spectra (Figure 5) have similar appearances as Org-I and Org-II are polymorphs. Furthermore, the resolution of these spectra indicates that absolute quantification is inaccessible by peak integration. This is demonstrated by the ^1^H wDUMBO spectrum of Org-I 70% shown in Figure 6a. None of the Org OD 14 ^1^H signals are well resolved except for the Org-I signal at ca. 4.5 ppm. The lack of distinct peaks for Org-II renders absolute quantification using peak integration impractical. Hirsh et al. showed that peak-integration-based quantification can be accomplished using a single peak.^35^ However, a calibration plot is required, leading to an increase in sample preparation and experimental time.

Using CRAMPS–MAR, we successfully quantified the Org-I 70% sample with ease (Figure 6a). The mixture and reference spectra were each acquired within 8 min, allowing for rapid quantification. Figure 6a shows a good agreement between the CRAMPS–MAR fit and the experimental spectrum. The fit results were found to be highly accurate, arriving within ±1 wt % of the sample weight values (Table 1). No obvious sample signals were observed in the residue spectrum. Moreover, the corresponding difference spectra (*m* – *c*_Org-I_*p*_Org-I_ and *m* – *c*_Org-II_*p*_Org-II_) mimic the pure component spectra, indicating that the pure component spectra were properly scaled and aligned (Figure 6b,c).

**Table 1:** The Weight Percent of Org-I in the Org-I/-II Mixture Determined by Sample Weight and CRAMPS–MAR^a^

method	Org-I
sample weight^b^	69.9 ± 0.4
CRAMPS–MAR^c^	70.3 ± 2.3

a All errors are expressed as 95% confidence intervals.

b Errors derived from the uncertainty associated with the analytical balance.

c Errors were propagated from the least-square fitting errors.

To estimate the quantification limit of CRAMPS–MAR for Org-I/-II mixtures, we have simulated a series of mixture spectra using the pure component spectra (Figure S14). According to our Pio/PioHCl results, CRAMPS–MAR can accurately quantify mixtures that display spectral features for the minor component. For Org-I and Org-II, the corresponding signals can still be seen when the mixture contains 10 and 85 wt % of Org-I, respectively. Thus, we estimate the quantification limit to be ca. 10 wt % for Org-I and ca. 15 wt % for Org-II. The experimental limit is likely slightly lower than our estimate since, as shown by our Pio 5% results, accurate quantification can be accomplished even when the minor component signals are not clearly observed in the mixture spectra. Our Org OD 14 results thus demonstrate the broad applicability of CRAMPS–MAR in quantifying solid-state structures of pharmaceuticals. In particular, accurate and calibration-free quantification can be accomplished even when analogous analyses cannot be realized using ultra-fast MAS with peak integration.

Spectral resolution plays a crucial role in a successful CRAMPS–MAR analysis. However, the ^1^H spectra of structurally complex APIs can be highly congested. As seen with Org-I/-II, the corresponding mixture spectrum can exhibit very little spectral features for the minor component. Moreover, spectral crowding will intensify as more components (> 2) are added into the mixture. Thus, we expect the performance of CRAMPS–MAR to decline as the analytes’ structures and/or the mixture becomes more complex. Nonetheless, the resolution of CRAMPS is continuously being improved.^49,51,53,70,71^ With the recent progress, we believe that CRAMPS–MAR can still advance, allowing complex, multi-component pharmaceutical mixtures to be quantified with ease.

## Conclusions

In this work, we proposed a novel SSNMR approach, CRAMPS–MAR, for rapid quantification of APIs. CRAMPS can provide high-resolution ^1^H SSNMR data with standard equipment, while MAR allows complex data to be effortlessly analyzed. By combining the two techniques, a rapid, straightforward, and sensitive quantification can be realized. First, quantification can be accomplished within minutes, which is significantly faster than the common ^13^C SSNMR approach. Second, CRAMPS–MAR data analysis is simple to conduct. Unlike the traditional peak-integration method, accurate CRAMPS–MAR analysis does not entail peak assignment. Third, the “fingerprinting” nature of CRAMPS–MAR allows for a more relaxed prerequisite on spectral resolution, thereby enabling a lower quantification limit.

By utilizing CRAMPS–MAR, we successfully quantified mixtures of Pio/PioHCl and Org-I/-II. Our results surpass those obtained using fast and/or ultra-fast MAS with peak integration. For Pio/PioHCl, CRAMPS–MAR provided a lower quantification limit by utilizing crowded spectral regions that are inaccessible for fast MAS with peak integration. Consequently, we were able to quantify mixtures with 5 wt % of Pio and 3 wt % of PioHCl, respectively. The corresponding results were extremely accurate, landing within ±1 wt % of the sample weight values. To demonstrate the robustness of CRAMPS–MAR, we used a polymorphic mixture of a steroid (Org OD 14). We found absolute quantification to be unfeasible by ultra-fast MAS with peak integration. This is attributed to the spectral complexity, which was unalleviated even when state-of-the-art equipment were employed. Remarkably, higher resolution was observed in our wDUMBO spectra, and CRAMPS–MAR accurately determined the composition of the Org-I/-II mixture. Thus, our results demonstrate the suitability of CRAMPS–MAR in previously unachievable quantification analyses. Although our study was conducted on API mixtures, the methodology described should be extendable to other complex mixtures. Further advances in the resolution of CRAMPS spectra will certainly lower the limitations and increase the applicability of CRAMPS–MAR. As such, we believe that CRAMPS–MAR will evolve into an indispensable tool in analytical sciences.

## Supporting Information Available

Pio, PioHCl, and Org OD 14 chemical structures; additional sample preparation details; additional experimental and data processing details; CRAMPS–MAR procedure schematic; CRAMPS–MAR phasing discussion; CRAMPS–MAR scaling procedure discussion; CRAMPS–MAR binning discussion; Pio/PioHCl sample compositions and the CRAMPS–MAR results; CRAMPS–MAR fits for Pio 97 to 5% samples; CRAMPS–MAR fits for Pio 10% with 6 min and 1 h acquisition time; simulated Org-I/-II mixture spectra; in-house MATLAB script used for CRAMPS–MAR (PDF)
