# Supplementary material for: Rapid Quantification of Pharmaceuticals via 1H Solid-State NMR Spectroscopy
Source: Anal Chem. 2022 Nov 23;94(48):16667–74. doi: 10.1021/acs.analchem.2c02905 (PMC9730298; doi:10.1021/acs.analchem.2c02905)
Supplement: Supplementary file 1 — ac2c02905_si_001.pdf [file ac2c02905_si_001.pdf]

Supporting Information

Rapid Quantification of Pharmaceuticals via ^{1}H Solid-State NMR Spectroscopy

Y. T. Angel Wong,[a] Ruud L. E. G. Aspers,[a] Marketta Uusi-Penttilä,[b] Arno P. M. Kentgens[a,*]

[a] *Institute for Molecules and Materials, Radboud University, Heyendaalseweg 135, 6525 AJ Nijmegen, The Netherlands*

[b] *Aspen Oss B.V., Kloosterstraat 6, 5349 AB, Oss, The Netherlands*

[*] Corresponding Author: Arno P. M. Kentgens (a.kentgens@nmr.ru.nl)

Table of Contents

Discussion S1. Mixture sample preparation procedure S5

Discussion S2. Additional SSNMR experimental and data processing details S6

Discussion S3. Phasing ^{1}H CRAMPS spectra for MAR S10

Discussion S4. Scaling and aligning the ^{1}H CRAMPS spectra for MAR S16

Discussion S5. Influence of spectral binning on CRAMPS-MAR S22

Figure S1. Chemical structures of Pio, PioHCl, and Org OD 14 S4

Figure S2. Schematic outlining the CRAMPS-MAR Procedure S9

Figure S3. In-phase and ± 1.5°(0th order) out-of-phase Pio spectra S12

Figure S4. Influence of 0th order phasing on CRAMPS-MAR fits S13

Figure S5. Influence of 1st order phasing on CRAMPS-MAR fits S14

Figure S6. In-phase and ± 60°(1st order) out-of-phase Pio spectra S15

Figure S7. ^{1}H wDUMBO spectra of Pio, PioHCl, and 70 % Pio acquired under comparable experimental conditions S19

Figure S8. CRAMPS-MAR fit via scale-by-difference and scale-by-reference S20

Figure S9. PioHCl spectra with various extends of binning S25

Figure S10. Influence of binning on the absolute error and 95 % confidence interval of CRAMPS-MAR fit S27

Figure S11. Influence of binning on CRAMPS-MAR analysis time S28

Figure S12. CRAMPS-MAR fit for Pio 97, 95, 90, 60, 10, and 5 % samples S30

Figure S13. CRAMPS-MAR fits for Pio 10 % with 6 min and 1 hr acquisition time S31

Figure S14. Simulated Org-I/-II mixture spectra S32

Table S1. Amounts of Pio and PioHCl in the Pio/PioHCl binary mixtures S5

Table S2. Amounts of Org-I and Org-II in the Org-I/-II binary mixtures S5

Table S3. ^{1}H wDUMBO acquisition parameters S7

Table S4. ^{1}H → ^{13}C CP/MAS inversion recovery acquisition parameters S8

Table S5. ^{1}H one-pulse acquisition parameters S8

Table S6. CRAMPS-MAR fit results using scale-by-reference and scale-by-difference S21

Table S7. Binning conditions used to construct the spectra in Figure S9. S26

Table S8. Pio/PioHCl sample compositions and the corresponding CRAMPS-MAR results S29

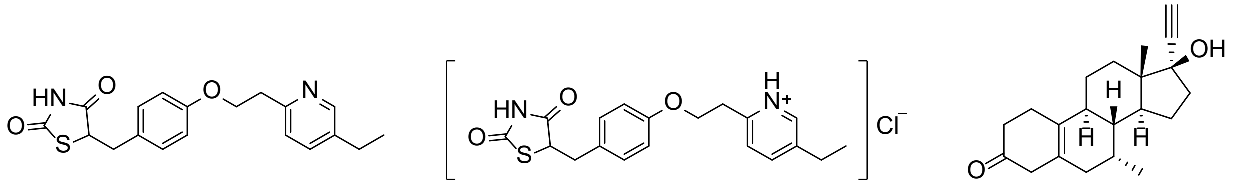

Figure S1. The chemical structures of (a) Pio, (b) PioHCl, and (c) Org OD 14.

Mixture Sample Preparation Procedure

To prepare the bicomponent mixtures, the respective components (*i.e.*, Pio and PioHCl, Org-I and Org-II) were weighted, then mixed for 12 to 48 hrs using a vortex mixer. The samples were manually inverted a few times during mixing to ensure homogeneity. The sample weights used to prepare the mixtures are given in Table S1 and S2.

Table S1. Amounts of Pio and PioHCl in the Pio/PioHCl binary mixtures.

Sample	Pio weight (mg)[a]	PioHCl weight (mg)[a]	Total weight (mg)[a]	Pio wt %[b,c]
Pio 97 %	259.2	7.9	267.1	97.0 ± 0.1
Pio 95 %	190.0	10.9	200.9	94.6 ± 0.2
Pio 90 %	45.0	6.1	51.1	88.1 ± 0.6
Pio 70 %	35.1	14.9	50.0	70.2 ± 0.6
Pio 60 %	30.1	20.3	50.4	59.7 ± 0.5
Pio 10 %	5.4	45.5	50.9	10.6 ± 0.4
Pio 5 %	10.5	193.0	203.5	5.2 ± 0.1

[a]All weights have an uncertainty of ± 0.1 mg. [b]wt % values are calculated from sample weights. [c]Errors are expressed as 95 % confidence intervals.

Table S2. Amounts of Org-I and Org-II in the Org-I/-II binary mixture.

Sample	Org-I weight (mg)[a]	Org-II weight (mg)[a]	Total weight (mg)[a]	Org-I wt %[b,c]
Org-I 70 %	47.4	20.4	67.8	69.9 ± 0.4

[a]All weights have an uncertainty of ± 0.1 mg. [b]wt% value calculated from sample weights. [c]Errors are expressed as 95 % confidence intervals.

Additional SSNMR Experimental and Data Processing Details

For the ^{1}H wDUMBO experiments, the sample spinning speed (11.1 kHz) was optimized based on the ^{1}H spectral resolution of glycine.[1] To minimize phase transient effects, and thus maximize spectral resolution, the probe tuning frequency was also optimized.[2] This was accomplished by carefully detuning the probe until the highest resolution ^{1}H wDUMBO spectrum was obtained for glycine. The transmitter offset frequency was adjusted to avoid overlap between rotary resonance frequency (RRF) lines and the sample signals while preserving spectral resolution.[3] The ^{1}H wDUMBO acquisition parameters are given in Table S3. The ^{1}H longitudinal relaxation time (T_1) was measured using linear-ramped cross-polarization (CP)/MAS inversion recovery with ^{13}C detection. The corresponding acquisition parameters are provided in Table S4.

For the ultrafast MAS ^{1}H SSNMR spectra, the experiments were performed with a one-pulse experiment. The corresponding acquisition parameters are provided in Table S5.

For MAR, binning was accomplished via equidistant binning, which gives uniform bin widths. Fitting was only conducted on spectral regions with sample signals, and the regions were represented by the same number of data points for a given bicomponent system (*i.e.*, Pio/PioHCl or Org-I/-II).

Table S3. ^1H wDUMBO acquisition parameters used for the samples in this study.

Parameter	Pio and PioHCl containing samples	Org-I and Org-II containing samples
Spectral width [kHz]	32.894737	32.894737
Recycle delay [s][a]	45	60
Number of scans	80 or 8	8
Number of data points acquired	512	1024
Excitation pulse (π/2) RF field strength [kHz]	113 – 116	122
DUMBO pulse length, 6π [us]	29.4	29.4
DUMBO pulse RF field strength [kHz][b]	112 - 114	120
DUMBO delay [us][c]	0.1	0.1

[a]Set to be at least 5 times the longest T_1 value. [b]As determined by the nutation curve of adamantane. [c]Delay between the DUMBO pulse and windowed acquisition.

Table S4. $^1H \rightarrow {}^{13}C$ CP/MAS inversion recovery acquisition parameters used for the samples in this study.

Parameter	PioHCl	Pio	Org-I	Org-II
Spectral width [kHz]	62.5	62.5	62.5	62.5
Recycle delay [s]	200	160	250	200
Number of scans	64	64	8	24
Number of data points acquired	2048	2048	3750	2048
Excitation pulse (π/2) RF field strength [kHz]	116	116	114	119
^{13}C CP pulse RF field strength [kHz]	64	64	63	60
^1H CP pulse RF field strength [kHz], linear-ramped	61 to 91	61 to 91	55 to 95	57 to 87
CP contact time [ms]	4	4	2.6	10
^1H SPINAL decoupling RF field strength [kHz]	100	100	103	100

Table S5. ^1H one-pulse acquisition parameters used for Org-I and Org-II containing samples.

Parameter	Org-I and Org-II
Spectral width [kHz]	250
Recycle delay [s]	0.5
Number of scans	1
Number of data points acquired	4000
Excitation pulse length [us]	0.6 for Org-I, 1.4 for Org-II

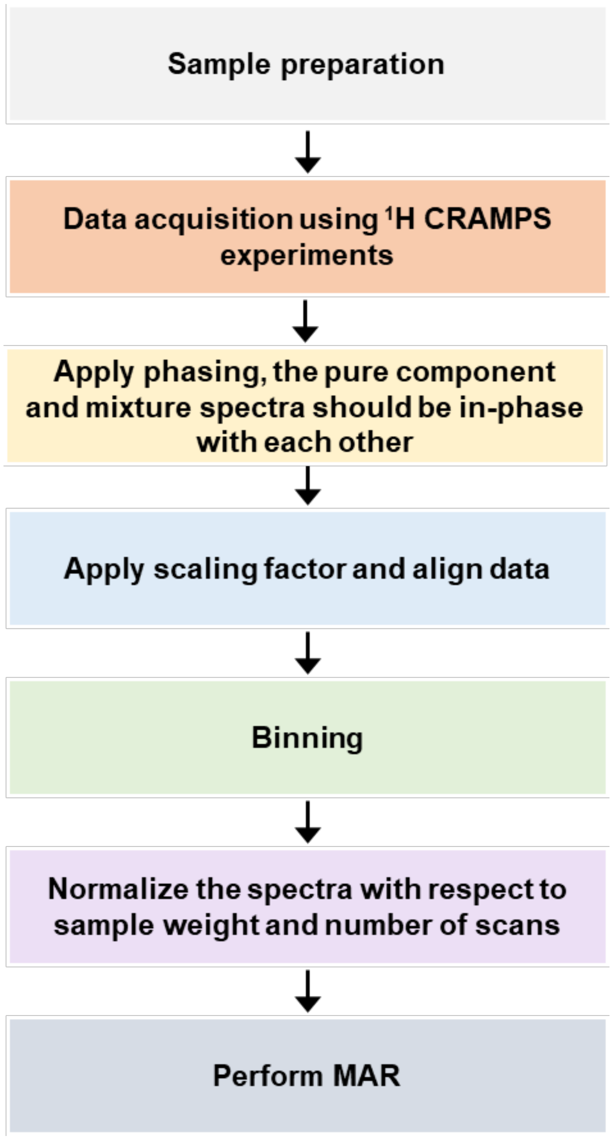

Figure S2. Schematic outlining the CRAMPS-MAR procedure.

Phasing ^{1}H CRAMPS spectra for MAR

For an accurate CRAMPS-MAR analysis, the pure component spectra must represent the mixture spectrum. Therefore, the spectra must be in-phase with each other. We have investigated the importance of phasing for CRAMPS-MAR using the Pio 90 % dataset. Successful CRAMPS-MAR quantification was achieved when the pure component and mixture spectra are in-phase (Figure S3a). The composition of Pio was found to be 87.2 to 89.0 wt %, agreeing with the composition determined by sample weight (87.5 to 88.7 wt %).

To examine the influence of 0th order phasing, the Pio spectrum was adjusted from in-phase with the PioHCl and mixture spectra to ± 3° out-of-phase. Figure S4 shows the CRAMPS-MAR results and the corresponding 95% CIs. As expected, the accuracy CRAMPS-MAR decreases as the spectra become more out-of-phase. At large phase deviations (ca. greater than ± 1.5°), the CRAMPS-MAR results become inaccurate. For instance, this is observed when the phase was modified by +3°, where the corresponding CRAMPS-MAR 95 % CI no longer encapsulates the Pio wt % from sample weight. The 95 % CI also increases as the spectra become more out-of-phase, indicating a decrease in precision. Nevertheless, CRAMPS-MAR is robust towards slight differences in 0th order phasing. This is demonstrated when the phase was varied by ± 1.5°. Figure S3 shows that the Pio spectra are visibly out-of-phase from the mixture spectrum. However, CRAMPS-MAR still provided an accurate quantification result. For example, when the phase is varied by -1.5°, the composition of Pio was determined to be 86.1 to 87.7 wt %, agreeing with the 87.5 to 88.7 wt % calculated from the sample weight. Moreover, the 95 % CIs were also comparable to those obtained from the in-phase spectra (0.8 to 1.1 wt % vs. 0.9 wt %), indicating

that slight changes in phasing does not influence fit precision. Thus, small deviations in 0th order phasing will not be detrimental to the CRAMPS-MAR results.

To investigate the influence of 1st order phasing, the Pio spectrum was adjusted from in-phase to ± 100° out-of-phase. Figure S5 shows the CRAMPS-MAR fit results and the corresponding 95 % CIs. As compared to 0th order phasing, CRAMPS-MAR results are less sensitive to 1st order phasing. When varied by ± 60°, the Pio spectrum is clearly out of phase from the mixture spectrum (Figure S6). Nonetheless, accurate quantification was still achievable via CRAMPS-MAR. For instance, when the phase was changed by -60°, the Pio composition was found to be 87.3 to 89.2 wt % via CRAMPS-MAR, in agreement with the 87.5 to 88.7 wt % calculated from sample weight. Moreover, the 95 % CIs were also comparable to that of the in-phase spectra (ca. 0.9 to 1.1 wt % vs. 0.9 wt %), indicating that the fit precision was not affected. Even when the phasing was adjusted by ± 100°, CRAMPS-MAR still provided accurate results, albeit a decrease in precision (Figure S5). Nevertheless, to ensure the best accuracy and precision, the pure component and mixture spectra should still be in-phase to both the 0th and 1st order. As CRAMPS-MAR is robust even when the spectra appear to be slightly out-of-phase, visual inspections can be used to evaluate the quality of the phasing. If the spectra appear to be in-phase, then the phasing is adequate for a CRAMPS-MAR analysis.

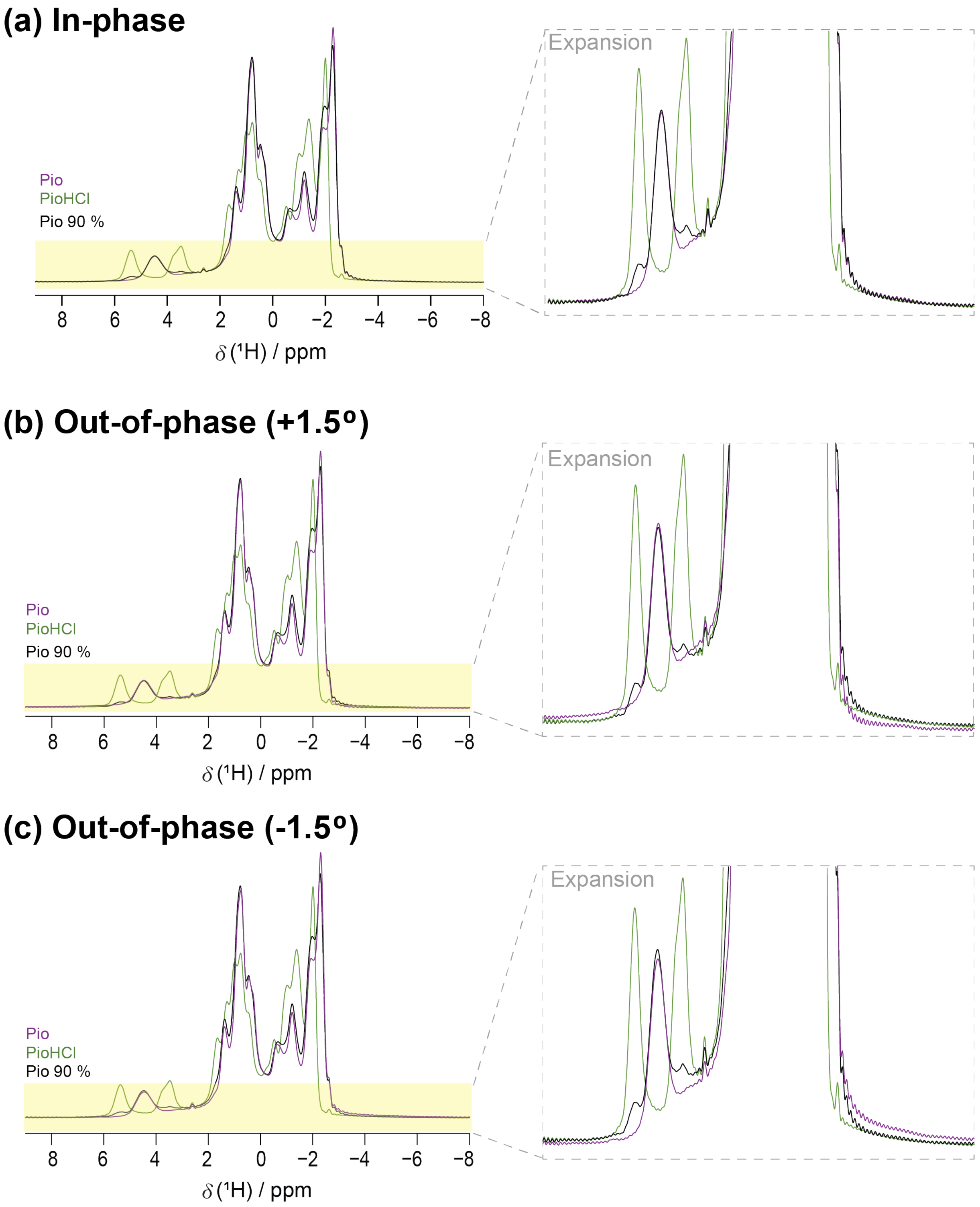

Figure S3. The Pio spectrum as (a) in phase and (b and c) out-of-phase with the PioHCl and Pio 90 % spectra. A 0th order phasing of +1.5^{o} and -1.5^{o} were applied to the in-phase Pio spectra to obtain the Pio spectra in (b) and (c), respectively. The pivot point for the phasing is at 0 ppm. No chemical shift scaling or referencing has been applied on the data.

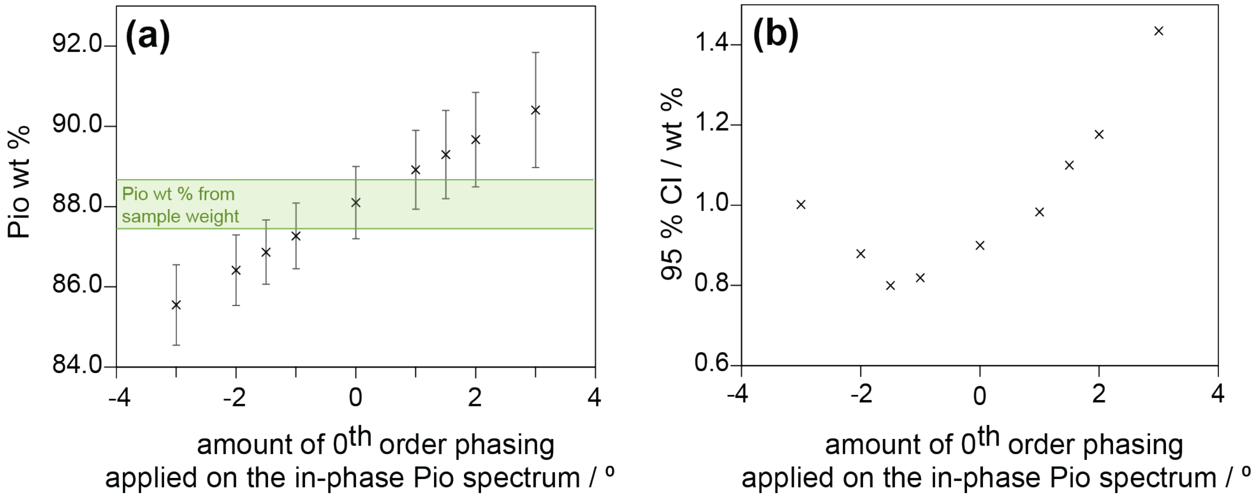

Figure S4. (a) The CRAMPS-MAR-predicted Pio wt % and (b) the corresponding 95 % CIs as a function of the amount of 0th order phasing applied on the Pio spectrum. 0° corresponds to the Pio spectrum being in-phase with the PioHCl and mixture spectra, while deviation from 0° corresponds to the Pio spectrum being out-of-phase with the PioHCl and mixture spectra. The Pio wt % obtained from sample weight is marked by the horizontal green bar.

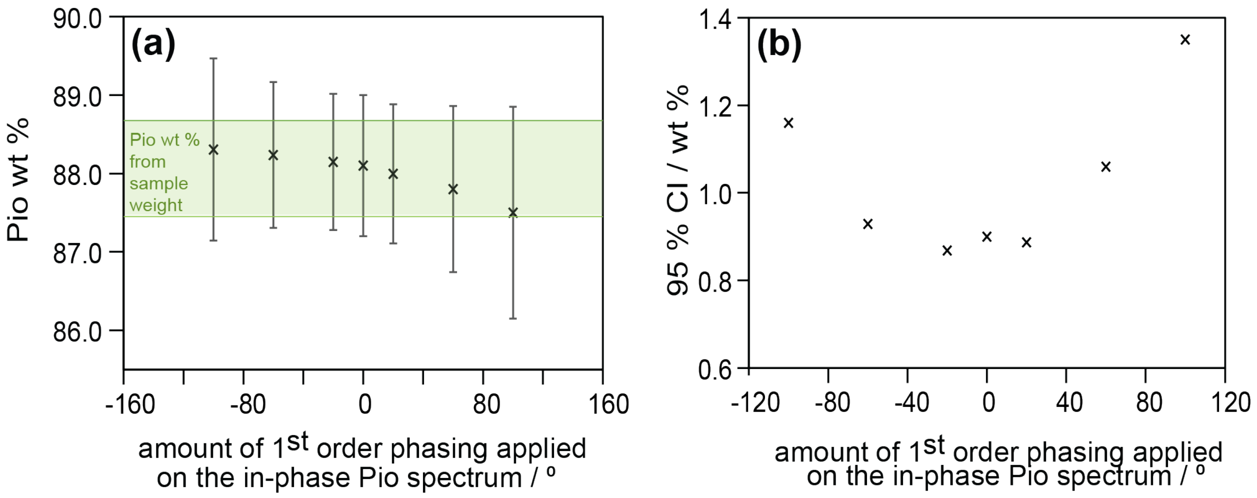

Figure S5. (a) The CRAMPS-MAR-predicted Pio wt % and (b) the corresponding 95 % CIs as a function of the amount of 1st order phasing applied on the Pio spectrum. 0° corresponds to the Pio spectrum being in-phase with the PioHCl and mixture spectra, while deviation from 0° corresponds to the Pio spectrum being out-of-phase with the PioHCl and mixture spectra. The Pio wt % obtained from sample weight is marked by the horizontal green bar.

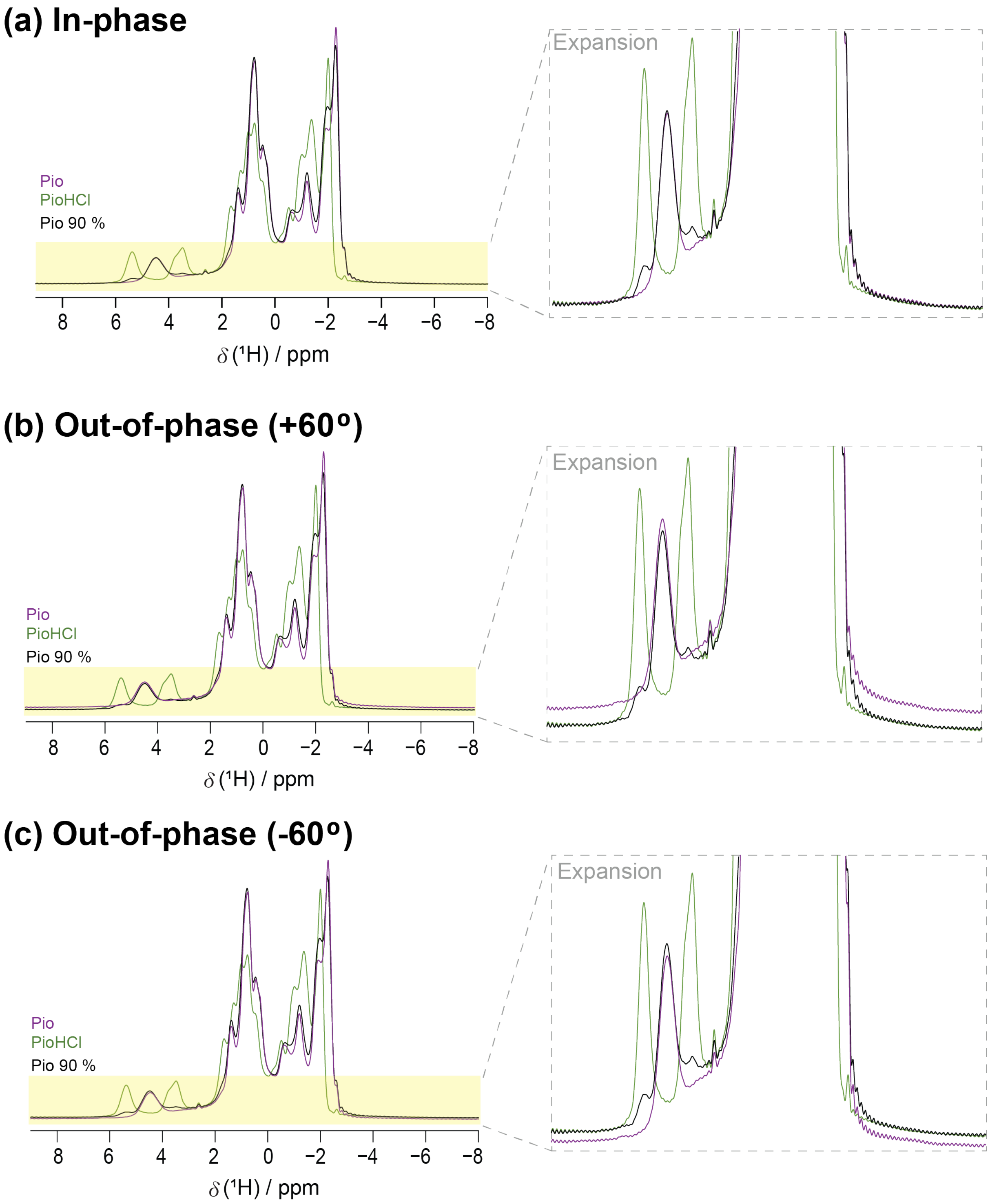

Figure S6. The Pio spectrum as (a) in phase and (b and c) out-of-phase with the PioHCl and Pio 90 % spectra. A 1st order phasing of +60° and -60° were applied to the in-phase Pio spectra to obtain the Pio spectra in (b) and (c), respectively. The pivot point for the phasing is at 0 ppm. No chemical shift scaling or referencing has been applied on the data.

Scaling and Aligning the ^{1}H CRAMPS spectra for MAR

For an accurate MAR fit, the pure component spectra (p_1, p_2, …, p_N) must represent the components in the mixture spectrum (m). For ^{1}H CRAMPS experiments, peaks positions can differ between spectra acquired under comparable experimental conditions due to small variations in the chemical shift scaling factors, σ.[2-4] This can be seen in the Pio 70 % data set (Figure S7). The left-most and right-most signals in the pure component spectra have a chemical shift difference of 7.36 ppm for PioHCl and 6.76 ppm for Pio. However, the chemical shift difference is 7.33 ppm for the PioHCl signals and 6.77 ppm for the Pio signals in the Pio 70 % spectrum. Thus, the pure component spectra must be scaled and aligned to the mixture spectrum.

To accurately scale and align a spectrum, 2 distinct spectral features are required as σ is applied linearly on the frequency axis (*i.e.*, σν, where ν = a frequency value).[4] Depending on the spectral resolution of m, different methods can be used for scaling and alignment. In the simplest case, m clearly displays at least 2 spectral features for each of the pure components. This is seen in the Pio 70 % mixture spectrum (Figure S7). Under these circumstances, p_1 to p_N can be directly scaled and aligned to m using the distinct spectral features.

In more congested spectra, m might not have enough spectral features for one of the components (component 1). Consequently, the spectrum of component 1 (p_1) cannot be directly scaled and aligned to m. For example, in the Pio 10 % spectrum, features of pure Pio is no longer clearly observed (Figure S8a). In these scenarios, we have developed two methods, scale-by-difference and scale-by-reference, to scale and align the pure component spectra of a bicomponent system.

In the scale-by-difference method, the pure component spectrum with at least 2 distinct spectral features (p_2) in m is first directly scaled and aligned. The same scaling and alignment factors are then applied on p_1 as an initial configuration. A weighted least-squares fit is applied on m, with a weight of 100% assigned to the well-resolved peaks of p_2. This fit provides an approximate contribution of p_2 to m, allowing a difference spectrum of m – c_2p_2 to be calculated. The difference spectrum should resemble p_1 and can therefore be used to accurately scale and align p_1. A CRAMPS-MAR fit is then performed using the fully scaled and aligned spectra. The procedure for scale-by-difference is demonstrated using Pio 10 % as an example (Figure S8b). First, PioHCl is directly scaled and aligned to Pio 10 % as PioHCl signals can be clearly observed at ca. 16 and 1 ppm. The same scaling and alignment factors are then applied on Pio. A weighted least-squares fit is performed with 100 % weight placed on the PioHCl peaks at ca. 16 and 12 ppm. The difference spectrum generated by m-$c_{pioHCl}p_{pioHCl}$ resembles p_{pio} and was used to rescale and align p_{pio}. A CRAMPS-MAR fit is then performed using p_{pio} and p_{pioHCl} that are properly scaled and aligned.

In the scale-by-reference method, a reference spectrum (m_R) is employed. m_R must exhibit at least 2 distinct spectral features for each pure component, allowing p_1, p_2, and m to be directly scaled and aligned to m_R. Since all spectra are scaled and aligned to m_R, they are also scaled and aligned to each other. The is analogous to secondary chemical shift referencing. Figure S8c shows scale-by-reference using Pio 10 % as an example. Pio 90 % was used as a reference. The Pio 90 % spectrum shows distinct Pio and PioHCl features at ca. 0.5, 12, 14, and 16 ppm. Thus, the PioHCl, Pio, and Pio 10 % spectra can be directly scaled and aligned to Pio 90 %. The resulting Pio and PioHCl spectra are therefore also scaled and aligned to Pio 10 %.

Based on the Pio 10 % data set, we found that scale-by-difference and scale-by-reference give comparable fit accuracy and precision (Table S6). The choice between scale-by-difference and scale-by-reference depends on the number of mixtures to be analyzed. If only one mixture is of interest, then scale-by-difference is the simpler solution. On the other hand, scale-by-reference is more straightforward if a series of mixtures is under investigation, and one of the mixtures can be used as a reference. Regardless of the method, the accuracy of the scaling and alignment can be verified post fitting. If scaling and alignment were performed correctly, the difference spectrum of $m – c_1p_1$, where c_1 is the weighting coefficient for p_1 in the CRAMPS-MAR fit, should resemble p_2 (see Figure 2 for example).

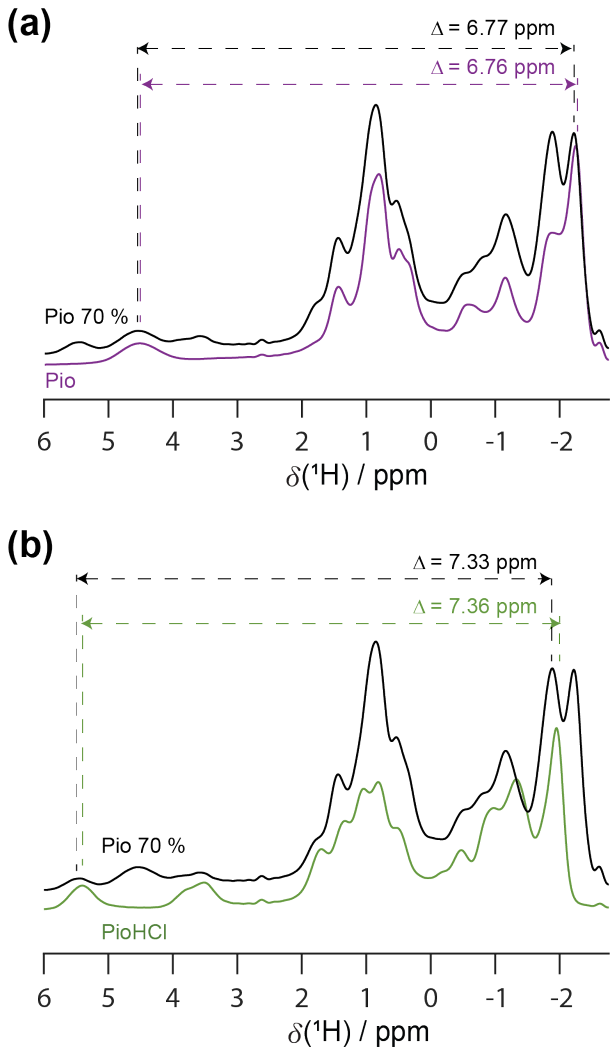

Figure S7. The ^{1}H wDUMBO spectra of PioHCl (green trace), Pio (purple trace), and Pio 70 % (black traces) acquired under comparable experimental conditions. No chemical shift scaling or referencing has been applied on the data.

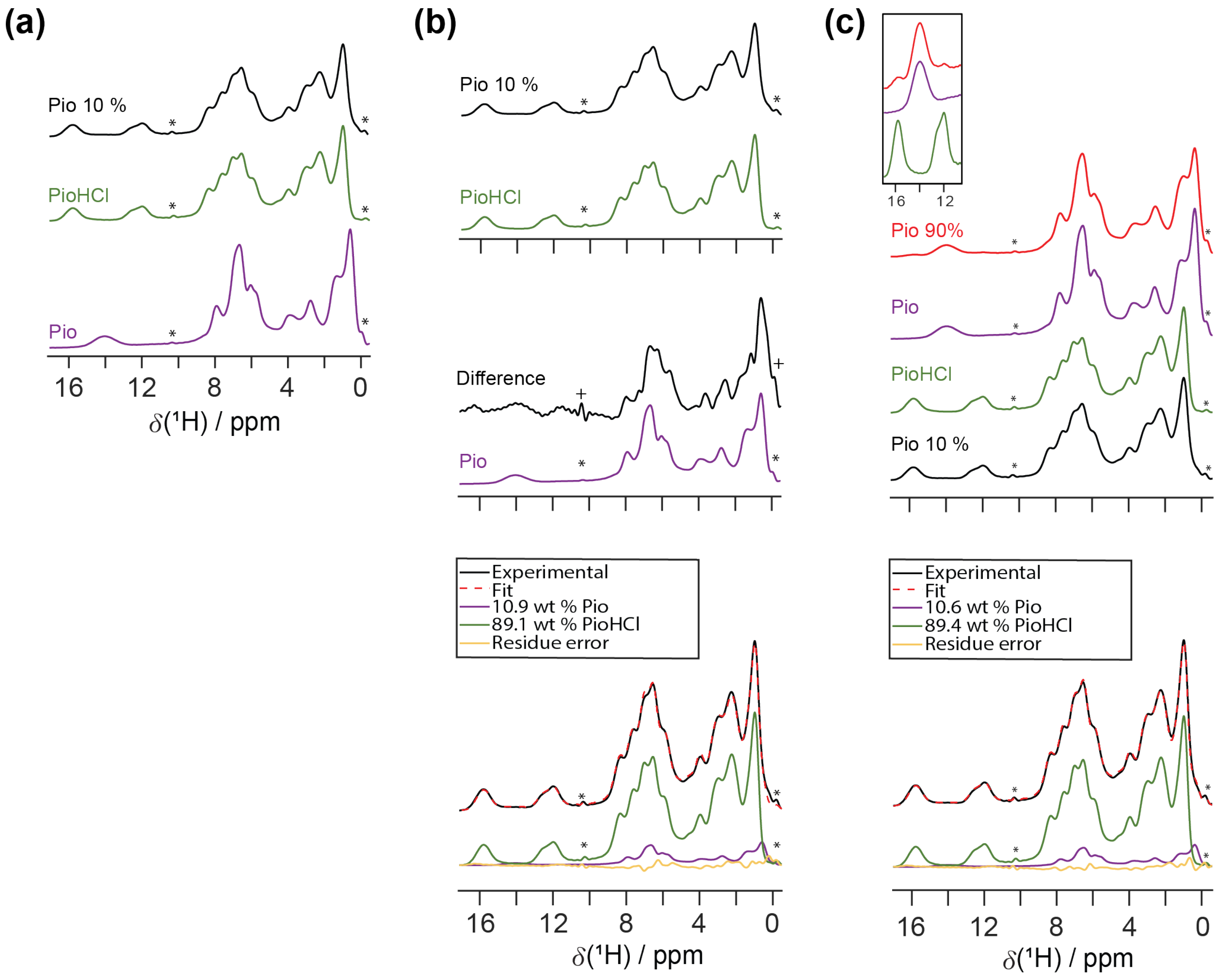

Figure S8. (a) The ^{1}H wDUMBO spectra of Pio 10 %, PioHCl, and Pio. (b) CRAMPS-MAR fit via scale-by-difference. The top panel shows the Pio 10 % spectrum, and the scaled and aligned PioHCl spectrum. The middle panel shows the difference spectrum calculated using m-$c_{pioHCl}p_{pioHCl}$ (black trace), and the Pio spectrum. The bottom panel shows Pio 10 % spectrum, the CRAMPS-MAR fit, and the individual fit components. (c) CRAMPS-MAR fit via scale-by-reference. The top panel shows the Pio 90 % spectrum (red trace) together with the Pio, PioHCl, and Pio10 % spectra that are scaled and aligned to Pio 90 %. Inset shows the expanded region from ca. 11 to 16.5 ppm for Pio 90 %, Pio, and PioHCl. The bottom panel shows Pio 10 % spectrum, the CRAMPS-MAR fit, and the individual fit components. Asterisks (*) denote RRF lines, and the crosses (+) denote artifacts from applying 0 weights on the RRF lines during fitting. The spectra for Pio 10 %, PioHCl, Pio, residue error, and CRAMPS-MAR fit are shown as black traces, green traces, purple traces, yellow traces, and red dotted traces, respectively.

Table S6. The wt % of Pio in Pio 10 % as obtained via sample weight and CRAMPS-MAR. For CRAMPS-MAR, two methods, scale-by-difference and scale-by-reference, were used for scaling and aligning the spectra.

Method	Pio
sample weight[a]	10.6 ± 0.4
scale-by-difference[b]	10.9 ± 0.7
scale-by-reference[b]	10.6 ± 0.7

[a]Errors derived from the uncertainty associated with the analytical balance. [b]Errors were propagated from the least-squares fitting errors.

Influence of Spectral Binning on CRAMPS-MAR

After applying the appropriate scaling and alignment factors on the spectra, the corresponding data points will occur at different frequencies between different spectra. Thus, the spectra must be binned. Data binning is frequently employed in chemometric analyses of NMR data to eliminate unwanted peak misalignments.[5-7] In data binning, the spectrum is divided into small sections (*i.e.*, bins), which is commonly accomplished by using bins of equal widths.[5, 6] The spectral area in each bin is then calculated, and the areas are used to represent the spectral intensities. Accordingly, multiple data points are combined into one via binning, and minor peak and/or data point misalignments can be corrected. Nonetheless, binning can drastically reduce the spectral resolution, which can impede accurate data analysis. Here, we investigated the influence of binning on the CRAMPS-MAR results and processing time. For consistency and comparability, the same set of raw ^{1}H wDUMBO data (PioHCl, Pio, and Pio 10 %) was employed for all our analyses presented in this section.

Figure S9 shows the pure PioHCl spectra with various extends of binning. Absolute chemical shift scaling or referencing with respect to glycine was not applied on the spectra, as these procedures are performed after the CRAMPS-MAR fit. The corresponding total number of data points and bin widths are provided in Table S7. Initially, the spectrum consists of 6999 data points, with a separation of 0.5 Hz between each data point. Consequently, the well-resolved signal at ca. 5.4 ppm, which has a full width at half maximum (FWHM) value of ca. 181.2 Hz, is represented by 1199 data points (Table S7). Upon binning, the apparent spectral resolution is maintained if the 5.4 ppm signal is denoted by at least 38 data points. Further binning to less than 38 data points decreases the resolution. In extreme cases, binning "distorts" the spectrum,

and the post-binned spectrum no longer represents the pre-binned spectrum. This can be seen in the spectrum where the signal at ca. 5.4 ppm is represented by 5 data points.

If binning decreases the apparent spectral resolution, the CRAMPS-MAR results will be inaccurate and imprecise. Figure S10a shows how binning influences the absolute error. The absolute error is calculated by |Pio wt % predicted by CRAMPS-MAR – Pio wt % calculated from sample weight|. If the 5.4 ppm signal of pure PioHCl is characterized by at least 38 data points, the absolute error remains at 0 wt %. However, if the signal is represented by less than 38 data points, the absolute error increases drastically from 0 to 9 wt %. This is due to a decrease in the apparent spectral resolution, lessening the number of features accessible for the least-squares fit. For example, the PioHCl spectrum displays ca. 12 features when the signal is represented by 1195 data points, but only ca. 5 features at 5 data points (Figure S9). Thus, drastic binning will decrease the CRAMPS-MAR fit accuracy.

Figure S10b shows that binning also influences the CRAMPS-MAR fit precision. As expected, the 95 % confidence interval slowly increases as the number of data points characterizing the 5.4 ppm peak of pure PioHCl decreases from 1195 to 76 However, a drastic increase in the 95 % confidence interval is observed in the region of 38 to 3 data points. This is again due to a decrease in the spectra's apparent resolution, resulting in less spectral features available for a precise fit. When an extreme amount of binning is performed, the 95 % confidence interval is expected to reach 0 since the least-squares equation can be solved analytically. This is seen when the PioHCl peak is represented by 1 data point. Based on our absolute error and 95 % confidence interval analysis, we conclude that binning is adequate if the apparent resolution is conserved. In the case of Pio/PioHCl systems, adequate binning is achieved if the bin widths are

at least ca. 10 times smaller than the signal's FWHM value (*e.g.*, 16.1 Hz for the 5.4 ppm signal with a FWHM value of 181.2 Hz).

We have also investigated the influence of binning on the CRAMPS-MAR analysis time. The pure component and mixture spectra were binned to give ca. 38 to 1195 data points for the 5.4 ppm peak of pure PioHCl. The post-binned spectra all provide adequate apparent resolution (Figure S9) and satisfactory fit results (Figure S10). The data analysis time includes binning, scaling and alignment, sample weight and number of scans normalization, and fitting. As expected, an increase in the number of data points results in an increase in data processing time (Figure S11). Nonetheless, the increase was found to be negligible. At 38 data points, the data took 3 seconds to analyze. Increasing the number of data points by a factor of ca. 31 only doubles the analysis time (6 seconds for 1195 data points). Thus, binning has a negligible influence on CRAMPS-MAR data analysis time. However, the number of data points should still be kept at a minimal to avoid any unnecessary increase in time.

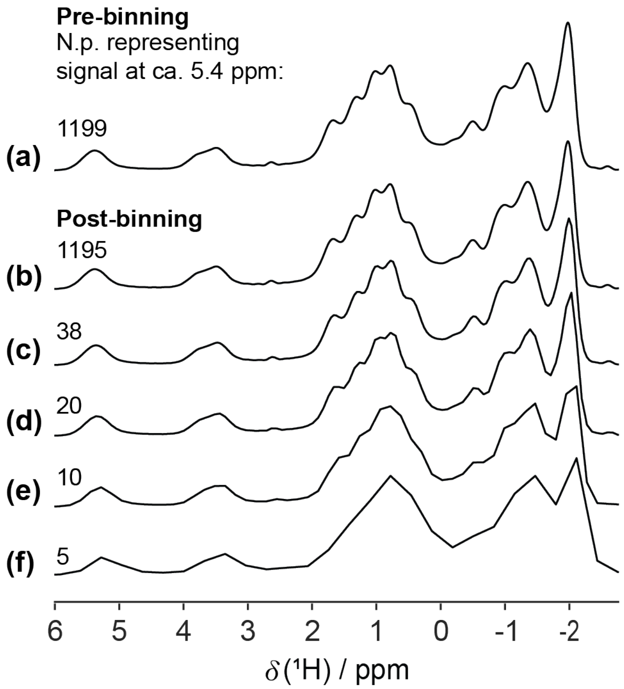

Figure S9. (a) The pre-binned ^{1}H wDUMBO SSNMR spectrum of pure PioHCl and (b-f) the corresponding post-binned spectra. The signal at 5.4 ppm is represented by different number of data points (n.p.) upon binning. No absolute chemical shift scaling or referencing with respect to glycine has been applied on the data.

Table S7. The total number of data points and the bin widths used to construct the spectra in Figure S9. The number of data points used to represent the 5.4 ppm signal in each spectrum is also provided.

Spectrum	Total number of data points	Bin width [Hz]	Number of data points used to represent the 5.4 ppm signal
a	6999	0.5[a]	1199
b	6979	0.5	1195
c	219	16.1	38
d	110	32.1	20
e	55	64.3	10
f	28	128.5	5

[a]Bin width corresponds to the frequency separation between each data point in the pre-binned spectrum.

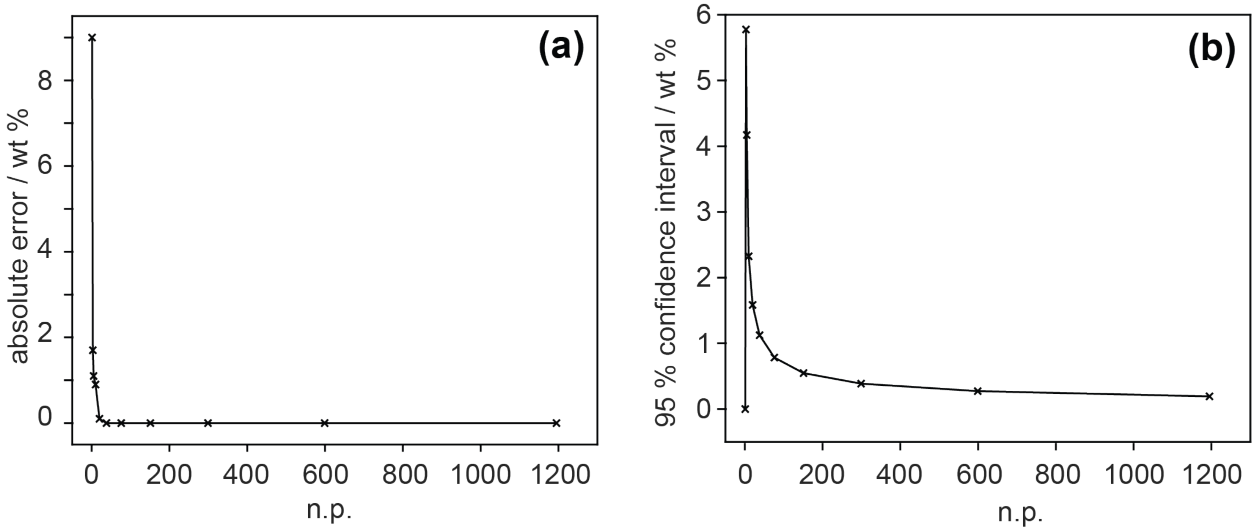

Figure S10. (a) The absolute error and (b) the 95 % confidence interval of the CRAMPS-MAR fit as a function of the number of data points (n.p.) representing the 5.4 ppm signal of pure PioHCl. For both (a) and (b), the data points are connected by a solid line to ease visualization.

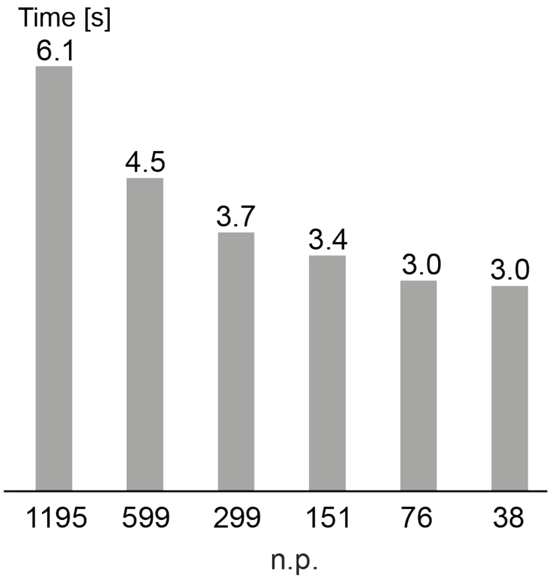

Figure S11. The CRAMPS-MAR analysis time as a function of the number of data points (n.p.) representing the 5.4 ppm peak of pure PioHCl.

Table S8. The wt % of Pio in the Pio/PioHCl binary mixtures determined by sample weight and CRAMPS-MAR. All errors are expressed as 95 % confidence intervals.

Sample	Pio 97 %	Pio 95 %	Pio 90 %	Pio 70 %	Pio 60 %	Pio 10 %	Pio 5 %
Sample weight[a]	97.0 ± 0.1	94.6 ± 0.2	88.1 ± 0.6	70.2 ± 0.6	59.7 ± 0.5	10.6 ± 0.4	5.2 ± 0.1
CRAMPS-MAR[b]	97.5 ± 0.9	94.6 ± 1.0	88.1 ± 0.9	69.4 ± 0.7	58.7 ± 1.0	10.6 ± 0.7	5.7 ± 0.5

[a]Errors derived from the uncertainty associated with the analytical balance. [b]Errors were propagated from the least-squares fitting errors.

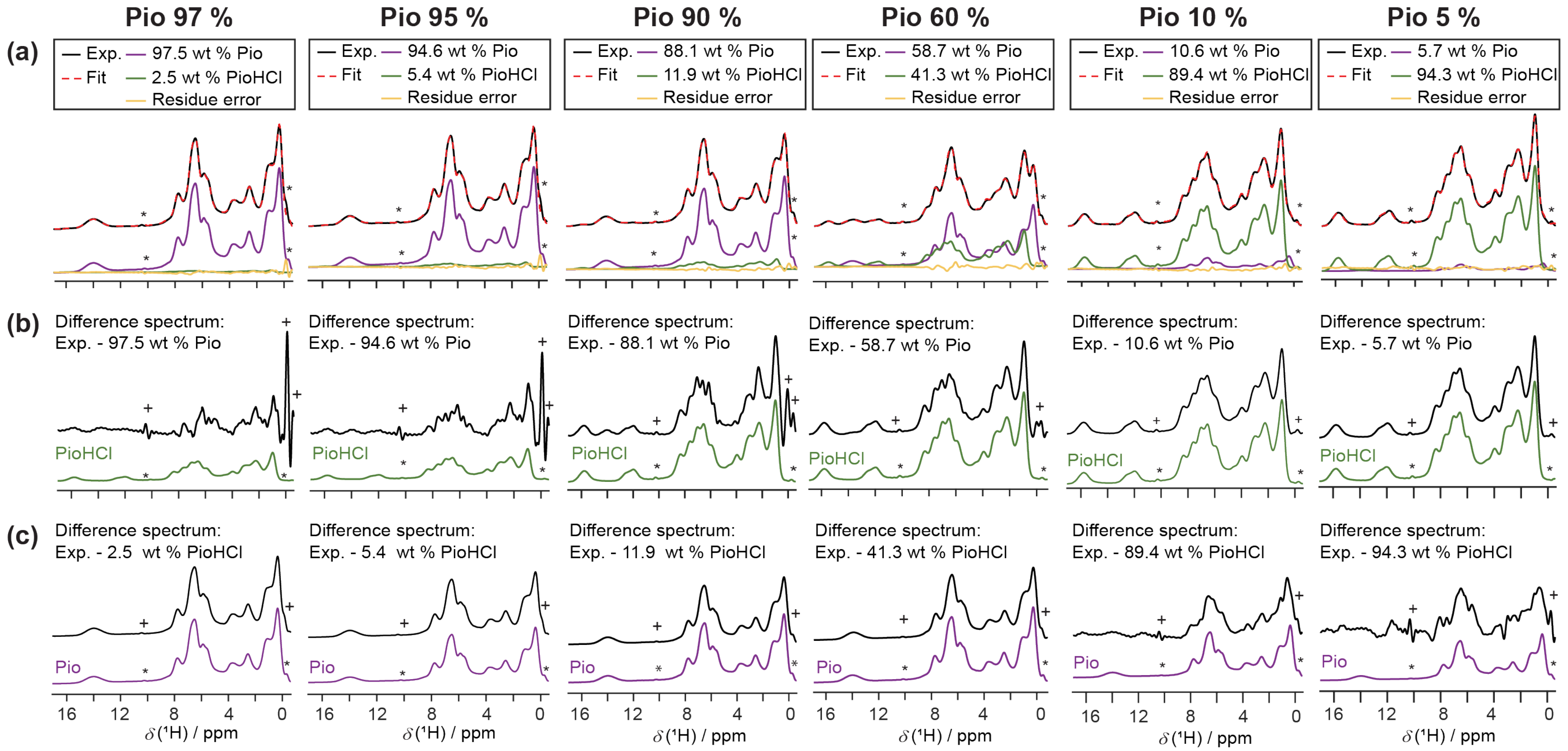

Figure S12. (a) The experimental ^{1}H wDUMBO spectrum (black traces) and the corresponding CRAMPS-MAR fit (red dotted traces) for Pio 97, 95, 90, 60, 10, and 5 %. The individual fit components (Pio: purple traces, PioHCl: green traces, residue error: yellow traces) are also provided. (b) The difference spectrum (black traces) obtained by subtracting the CRAMPS-MAR-predicted Pio contribution from the experimental spectrum. The pure PioHCl spectrum (green traces) is shown for reference. (c) The difference spectrum (black traces) acquired by removing the CRAMPS-MAR-predicted PioHCl spectrum from the experimental spectrum. The pure Pio spectrum (purple traces) is also provided for reference. Asterisks (*) denote RRF lines, crosses (+) denote artifacts from placing 0 weights on the RRF lines during the least-squares fit.

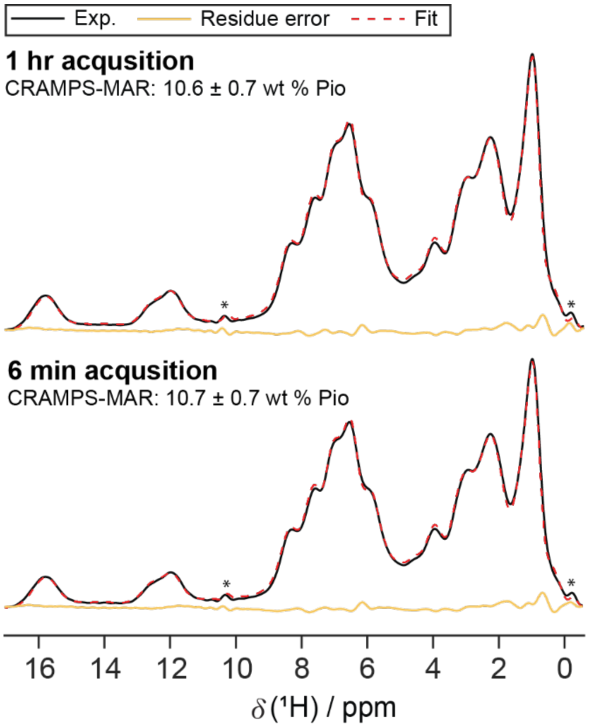

Figure S13. The ^{1}H wDUMBO spectra (black traces) of Pio 10 % acquired in 1 hour (top) versus 6 minutes (bottom). The corresponding CRAMPS-MAR fits (red dotted traces) and the residue error of the fits (yellow traces) are also provided. Asterisks (*) denote RRF lines.

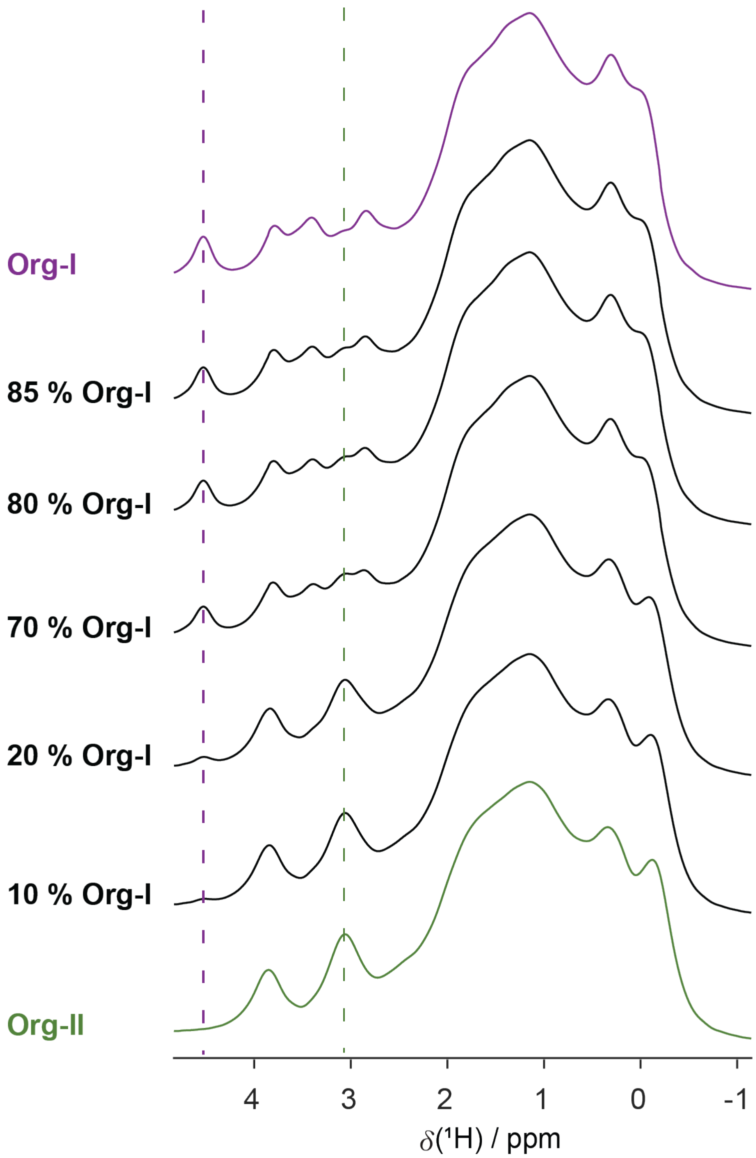

Figure S14. The experimental ^{1}H wDUMBO spectra for Org-I (purple trace) and Org-II (green trace), and the simulated spectra for Org-I/-II mixtures with 10 to 85 wt % Org-I (black traces). The simulated spectra were constructed using the pure component spectra via (x)Org-I spectrum + (1-x)Org-II spectrum, where x = 0.10, 0.20, 0.70, 0.80, and 0.85, respectively. Purple and green dotted lines denote the spectral features of Org-I and Org-II, respectively, that can be observed in the mixture spectra.

References

(1) Lesage, A.; Sakellariou, D.; Hediger, S.; Eléna, B.; Charmont, P.; Steuernagel, S.; Emsley, L. *J. Magn. Reson.* **2003**, *163*, 105-113.
(2) Brouwer, D. H.; Horvath, M. *Solid State Nucl. Magn. Reson.* **2015**, *71*, 30-40.
(3) Paruzzo, F. M.; Emsley, L. *J. Magn. Reson.* **2019**, *309*, 106598.
(4) Vega, A. J. *J. Magn. Reson.* **2004**, *170*, 22-41.
(5) Emwas, A.-H.; Saccenti, E.; Gao, X.; McKay, R. T.; Martins dos Santos, V. A. P.; Roy, R.; Wishart, D. S. *Metabolomics* **2018**, *14*, 31.
(6) Smolinska, A.; Blanchet, L.; Buydens, L. M. C.; Wijmenga, S. S. *Anal. Chim. Acta.* **2012**, *750*, 82-97.
(7) Worley, B.; Powers, R. *Acs Chem. Biol.* **2014**, *9*, 1138-1144.
